# People, plants and health: a conceptual framework for assessing changes in medicinal plant consumption

**DOI:** 10.1186/1746-4269-8-43

**Published:** 2012-11-13

**Authors:** Carsten Smith-Hall, Helle Overgaard Larsen, Mariève Pouliot

**Affiliations:** 1 Department of Food and Resource Economics, Faculty of Science, University of Copenhagen, Rolighedsvej 23, Frederiksberg C, 1958, Denmark

**Keywords:** Medicinal plants, Traditional medicine, Health, Global

## Abstract

**Background:**

A large number of people in both developing and developed countries rely on medicinal plant products to maintain their health or treat illnesses. Available evidence suggests that medicinal plant consumption will remain stable or increase in the short to medium term. Knowledge on what factors determine medicinal plant consumption is, however, scattered across many disciplines, impeding, for example, systematic consideration of plant-based traditional medicine in national health care systems. The aim of the paper is to develop a conceptual framework for understanding medicinal plant consumption dynamics. Consumption is employed in the economic sense: use of medicinal plants by consumers or in the production of other goods.

**Methods:**

PubMed and Web of Knowledge (formerly Web of Science) were searched using a set of medicinal plant key terms (folk/peasant/rural/traditional/ethno/indigenous/CAM/herbal/botanical/phytotherapy); each search terms was combined with terms related to medicinal plant consumption dynamics (medicinal plants/health care/preference/trade/treatment seeking behavior/domestication/sustainability/conservation/urban/migration/climate change/policy/production systems). To eliminate studies not directly focused on medicinal plant consumption, searches were limited by a number of terms (chemistry/clinical/in vitro/antibacterial/dose/molecular/trial/efficacy/antimicrobial/alkaloid/bioactive/inhibit/antibody/purification/antioxidant/DNA/rat/aqueous). A total of 1940 references were identified; manual screening for relevance reduced this to 645 relevant documents. As the conceptual framework emerged inductively, additional targeted literature searches were undertaken on specific factors and link, bringing the final number of references to 737.

**Results:**

The paper first defines the four main groups of medicinal plant users (1. Hunter-gatherers, 2. Farmers and pastoralists, 3. Urban and peri-urban people, 4. Entrepreneurs) and the three main types of benefits (consumer, producer, society-wide) derived from medicinal plants usage. Then a single unified conceptual framework for understanding the factors influencing medicinal plant consumption in the economic sense is proposed; the framework distinguishes four spatial levels of analysis (international, national, local, household) and identifies and describes 15 factors and their relationships.

**Conclusions:**

The framework provides a basis for increasing our conceptual understanding of medicinal plant consumption dynamics, allows a positioning of existing studies, and can serve to guide future research in the area. This would inform the formation of future health and natural resource management policies.

## Background

Medicinal plants, defined as plants used for maintaining health and/or treating specific ailments, are used in a plethora of ways in both allopathic and traditional systems of medicine in countries across the world. Even people using only allopathic medicine throughout their lives are likely to be somewhat medicinal plant reliant as 20-25% of drugs prescribed are plant derived [1]. There is thus a medicinal plant reliance continuum: from people who consume solely allopathic medicine to users having no alternative to using medicinal plants for a majority of their health care treatments. It is unfortunately not possible to even roughly estimate the absolute number of people, or the frequency of medicinal plant use, at different locations along the medicinal plant reliance continuum. Official statistics on medicinal plant trade and consumption are scant and not very informative as medicinal plant products are often part of the informal economy (the part of the economy not monitored by the government, taxed or included in national statistical estimates such as the gross national product) and thus not recorded, or recording aggregates medicinal plants with other items. Throughout this paper we use the term “consumption” in the economic sense: use of medicinal plants by consumers or in the production of other goods. The term is not used in any medical sense, e.g. to denote oral administration of a drug.

The World Health Organization has estimated that 80% of the world’s population relies solely or largely on traditional remedies for health care [2] and there is speculation that more than two billion people may be heavily reliant on medicinal plants [3]. Although considerable uncertainty surrounds these often cited figures, there is no doubt that medicinal plants play an important role in the livelihoods and welfare of a vast number of people in both developed and developing countries. The importance of medicinal plants in health care is increasingly recognized in the health sector as exemplified by discussions of the role of traditional medicine in contributing to achieving the Millennium Development Goals (MDG), three of which are directly health related [4], and by work towards European harmonized criteria for the assessment of herbal medicinal products [5,6]. When evaluating or developing nominal and functional health policies, it is crucial to understand the current role of medicinal plants and, in order to be able to assess the impacts of policy changes, to understand who is dependent how on medicinal plants. Will consumption increase in some locations and decrease in others? Should care be taken to reach certain groups of people? Health policies are only rarely integrated or coordinated with other sector policies (such as agricultural or environmental policies) with the result that health investments are narrowly confined to the health sector (e.g. [7]). Increased attention to medicinal plant consumption and its dynamics may contribute to the development of collaboration across the natural resources and health sectors, resulting in more comprehensive and efficient health policies.

Perhaps as a consequence of the ubiquitous worldwide use of medicinal plants, information on medicinal plant consumption is scattered across a wide range of disciplines and sectors, and there is no structured overview of state-of-knowledge. We argue that this impedes the systematic consideration of plant-based traditional medicine in national health care systems in many countries, although some notable examples of integration of herbal medicine into national health legislations exist (e.g. the European Directive on Traditional Herbal Medicinal Products). The objective of this paper is to improve our knowledge of medicinal plant consumption. We argue that there are many reliance dimensions linking humans and medicinal plants, and we use this as the starting point to identify the main groups of medicinal plant users and the main types of benefits they derive from medicinal plant usage. We then proceed to identify the factors determining medicinal plant consumption patterns and structure these in a conceptual framework. In other words, we address the two questions: (i) in what ways and to whom are medicinal plants important, and (ii) what factors determine medicinal plant consumption?

## Methods

The global peer-reviewed literature on medicinal plant use patterns and factors influencing these provided the foundation for outlining main users and benefit types. The large amount of relevant literature is found across many disciplines, for example ethnobotany, geography, anthropology and medicine, and the initial search was thus broad using search terms that would be most likely to generate studies that included medicinal plant consumption related aspects. We initially focused on the term traditional^a^ medicine and traced its history. This term was initially known as “primitive” medicine studied by anthropologists in third world countries. After World War II the term was succeeded by “peasant” and “folk” medicine, then “rural” medicine [8], and now the term in vogue is “traditional” (e.g. [9,10]). Traditional medicine is increasingly consumed in western countries, where it is commonly called “alternative/complementary/holistic/herbal/indigenous/integrative/native/natural/non-toxic/oriental/unconventional” and “fringe/non-traditional/unproven/unscientific”. It excludes what has been termed “allopathic/conventional/mainstream/modern/orthodox/western”. In our literature search we focused on the main terms used historically (folk/peasant/rural/traditional), terms that are of recent importance (ethno/indigenous/CAM) with the addition of terms that are entirely medicinal plant based (herbal/botanical/phytotherapy). We searched PubMed and the Thomson Reuters Web of Knowledge, with no language restrictions, and combined each of the search terms with other terms (medicinal plants/health care/preference/trade/treatment seeking behavior/domestication/sustainability/conservation/urban/migration/climate change/policy/production systems) to focus on studies including aspects of medicinal plant use and consumption. To eliminate studies that are not directly focused on medicinal plant use, such as chemical studies on plant constituents or clinical studies, the search was limited by a number of terms (chemistry/clinical/in vitro/antibacterial/dose/molecular/trial/efficacy/antimicrobial/alkaloid/bioactive/inhibit/antibody/purification/antioxidant/DNA/rat/aqueous). The search was last updated in August 2012. Taking into account 203 overlapping references in the two databases, a total of 1940 references were identified. A manual screening of the abstracts further eliminated 1295 references that were ethnobotanical descriptions (446) (only studies containing information directly relevant to the conceptual framework was included, e.g. a study documenting that a particular product from a particular species is used to treat a particular ailment in a particular location does not add to or deduct from the framework), concerned with safety and efficacy (239), veterinary medicine (39) or in other ways not relevant to the topic (574). The key term search thus yielded a total of 645 relevant documents. This formed the basis of the conceptual framework; as this emerged inductively additional targeted literature searches were undertaken to further clarify factors and links at especially the international and national levels. This identified an additional 92 documents. To avoid excessive referencing, the references included in the text are generally peer-reviewed reviews, comparative studies, and illustrative case studies. The full list of references, including how each reference is linked to the conceptual framework, is available in the Additional file 1: Appendix.

## Results

### How and to whom are medicinal plants important?

We distinguish three main types of benefits accruing from medicinal plant use: consumer, producer and society-wide benefits. Based on an existing typology [11], we classify users in four main groups: 1. Hunter-gatherers, 2. Farmers and pastoralists, 3. Urban and peri-urban (residing in areas between suburbs and rural areas) people, and 4. Entrepreneurs.

#### Types of benefits

*Consumer* benefits are (typically non-monetary) indirect benefits accruing from consumption of medicinal plant products, either raw or in processed form (e.g. [12,13]). Benefits are derived through both maintenance of health and treatment of illnesses. Quantification of these benefits is difficult but they may constitute the most important type of benefit in value terms. For example, it has been estimated that more than 50% of people facing illness in a rural setting in Burkina Faso consumed traditional medicine [14]. However, huge variation in the importance of consumer benefits across user groups and countries is to be expected.

*Producer* benefits are understood as (typically monetary) direct benefits from production of and trade in medicinal plants, plant based medicines, and plant based medicine services such as those provided by traditional medicine therapists (e.g. [15,16]). Benefits include harvester income from sale of medicinal plants and income to economic agents along the marketing chain where value-addition takes place, e.g. through transport and processing. Individual income levels range from marginal to substantial.

*Society-wide* benefits include employment opportunities in the medicinal plant based trade and industry, from processing to retailers and health care providers, as well as government revenues from medicinal plant related taxes (e.g. harvesting licenses, transport permits, custom duties and value-added tax). Trade may be of national economic importance [17]. In countries where conventional health care systems fail to reach or under-serve many people (e.g. [18]), traditional plant based medicines, by making health care (more) available and affordable, may result in a more healthy labour force with economy-wide productivity gains; this could be a major, not yet quantified, benefit.

#### Main user groups and associated medicinal plant benefits

**Figure 1:**
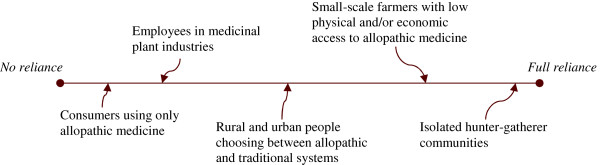
The medicinal plant reliance continuum and examples of reliance.

The huge differences in medicinal plant reliance between user groups [19] are visualized in the medicinal plant reliance continuum (Figure 1). The number of people at either end of the continuum is likely to be small: (i) at one extreme, few people use no medicine at all or only allopathic medicine not derived from plants, and (ii) at the other extreme, people entirely dependent on traditional medicine, such as in isolated hunter-gatherer communities, may have access to treatments based on minerals, animals and rituals (e.g. [20]).

*Hunter-gatherers* have strong cultural attachment to the environment and usually remain relatively isolated, having limited contact with market economies. They primarily rely on hunting and gathering or shifting cultivation and they are among the poorest of the poor (e.g. [21]). The relative number of people in this group is limited. Consumer benefits are generally very important for this user group. Hunter-gatherer communities are often remote and hence have the least access to public health care (e.g. [22,23]). This, combined with the cultural importance of the environment to those communities, usually leads to a relatively high reliance on medicinal plants for subsistence use.

*Farmers and pastoralists* typically occupy the landscape between forests and towns/cities and are engaged in subsistence and/or commercial agriculture, including animal husbandry. There is huge variation in this group ranging from landless farmhands to smallholders to large industrial and green revolution farmers [24]; the relative number of people in the group is high. Producer benefits to most small and medium scale farmers and pastoralists in developing countries from the production or collection of medicinal plants are limited due to a lack of access to technologies and exploitative market structures [25,26]. The degree of consumer benefits will typically vary with physical and economic access to the public health infrastructure [11,27-29], e.g. large farmers will have better economic access than landless (but see [30] for documentation of a positive correlation between wealth and the use of traditional medicine).

*Urban and peri-urban* people in developing and developed countries exhibit different medicinal plant consumption patterns. In developing countries, where the proportion of people living in urban areas is rising [31], the group includes a large number of poor that have migrated from rural areas to become part of the informal urban or peri-urban sector, and a smaller middle class with jobs in the formal sector (e.g. [32]) – though there are important exceptions, e.g. the large and rapidly expanding middle class in China. Medicinal plant consumption varies with factors such as income and access to public health facilities [27,33], ethnicity and gender [34] and ethnobotanical knowledge [35]. In developed countries, the main distinction is between the relatively poor and the well-off, where higher income [36] but also lack of access to modern treatment [37] predicts use of traditional medicine. The use of traditional medicine to prevent illness is apparently common [38] and persistent [36]. Generally, a high frequency of traditional medicine use is reported among migrants in developed countries [39,40].

*Entrepreneurs* are individuals who seek to capitalize on potentially profitable endeavours often associated with some degree of risk taking. They include actors along the medicinal plant marketing chain (traders, wholesalers, retailers), processors varying from small rural-based distillation units to huge urban based factories serving international markets, and health service providers such as traditional healers and general practitioners (e.g. [16,22,41-43]). The relative number in this group is limited but their functions are essential to make medicinal plants available to consumers. While producer benefits are intrinsically linked to entrepreneurs, consumer benefits depend on entrepreneurs’ access to public health facilities and levels of income.

### Determinants of medicinal plant consumption: A conceptual framework

**Figure 2:**
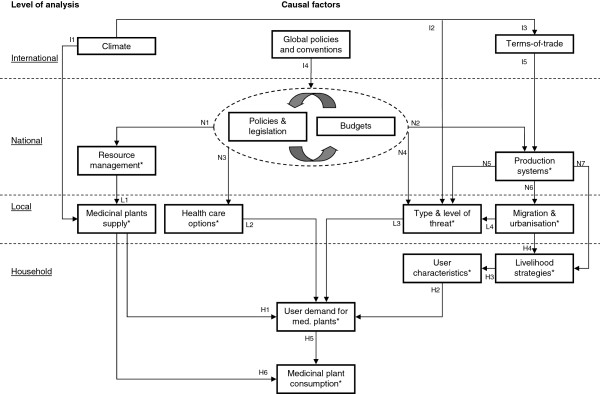
Conceptual framework for analyzing changes in medicinal plant consumption.

Analyzing medicinal plant consumption is very complex: there is a huge number of medicinal plant species from a large variety of habitats under different forms of management. They are used by different types of users in a vast number of preventive and curative treatments and offered by discrete types of therapists. To enable a systematic approach to understanding medicinal plant consumption, we here present a conceptual framework (Figure 2) focusing on the factors influencing the supply and demand of medicinal plants. There are four spatial levels of analysis: international, national, local, and household. At each level, three to four main factors and links (indicated by arrows) between factors are identified; each link is assigned a unique number (used when describing the links below). Note that arrows do not indicate simple uni-directional causalities, e.g. climate change may result in change in species composition in a forest which may simultaneously diminish the supply of one medicinal plant species while increasing the supply of another (link I1); impact is site and species specific. Also note that there are different types of impacts. Direct impacts are caused by physical or biological factors that influence medicinal plant consumption without interacting with social systems or other mechanisms, e.g. the direct impact of climatic changes on medicinal plant supplies through changed growth conditions. Indirect impacts are effects from economic, socio-political, institutional, demographic, technological and cultural activities that only influence medicinal plant consumption through other mechanisms, e.g. construction of roads into forest areas supplying medicinal plants (N1). Indirect impacts are site specific. Derived impacts are economy-wide and not restricted to particular sites, e.g. the impact of increased budgets for pluralistic national health care systems on health care options (N3). Finally, it should be noted that predicted changes in variables at national level and below are relative, i.e. influenced by the pre-existing situation. For instance, changes in medicinal plant supply are influenced by pre-existing factors such as the reproductive morphology of a species (I1).

In the following, each analytical level is explained, including a description of each causal factor and the linkages between factors. We highlight important assumptions and gaps in knowledge.

#### International level

There is now near unanimous agreement that anthropogenic greenhouse gas emissions will change the Earth’s climate [44]. Climate change will directly affect medicinal plant supply through changes in habitat structure and/or plant species composition (I1) [45] and may also change terms-of-trade (I3) as the relative cost of producing different items changes between countries. For instance, it has been predicted that climate changes will lead to decreasing average crop yields in developing countries and increased yields in developed countries [46]. Terms-of-trade are influenced by a large number of factors, including international flows of labour and capital and international commodity prices, which may all impact on the structure of national production systems (I5) (e.g. [47]). Climate change is also expected to affect human health (I2), mainly adversely, e.g. through an increase in vector-borne infectious diseases and extreme weather events (e.g. [48,49]). Finally, global policies and international multilateral conventions, such as the Convention on Biological Diversity and the Agreement on Trade-Related Aspects of Intellectual Property Rights, influence national policies, legislation, and budgets (I4) with impacts on both medicinal plant supply and demand. For example, international research on potential herbal medicines may be discouraged by national protectionist policies formulated to prevent biopiracy [50,51].

#### National level

Policies and budgets influence the supply of medicinal plants through decisions affecting resource management (N1). For example, enhancement of productivity can take place through resource management aiming at increasing the production area, protecting degraded production areas, and introducing more efficient technologies. Conversely, decreases in productivity result from elimination or degradation of production units, e.g. through policies promoting the expansion of agriculture in forests (e.g. [52,53]). Indirect impacts also arise from national allocations to infrastructure development and maintenance, including for roads that may indirectly impact the resource base through deforestation [54-56]. In many developing countries forest areas are presently shrinking and the forest quality deteriorating [57] leading to a reduction in the medicinal plant resource base. This may be exacerbated by commercialization of medicinal plants (e.g. [58]). There are presently only few attempts at increasing the production of medicinal plants [59]. If overexploitation of the natural medicinal plant resource persists higher prices will lead to lower consumption, unless the resource is domesticated [60].

Production systems refer to the general production structure in a country, including both traded and non-traded production in both urban and rural sectors; this structure determines land use patterns. Policies and budgets may (intentionally or unintentionally) directly influence the type, size and geographical location of production systems (N2). For instance, the 1999 Brazilian currency devaluation combined with an international price increase of soybeans and beef and control of hoof and mouth disease led to large scale replacement of savannah woodland, known to supply medicinal plants [61], by soybean and cattle in central-west Brazil [54]. Production systems are constantly changing, e.g. in response to subsidy programs or new or collapsing markets [62]. Changes in production systems in turn, through both pull and push factors, influence patterns of migration and rates of urbanization (N6). For instance, the collapse of the agricultural sector in El Salvador during the civil war led to large scale internal and international migration, including rapid growth in the capital city [47]. In the 1960s many Nepalese farmers moved from hill areas to lowlands in response to overcrowding and stagnant agricultural productivity (push) in the hills, and eradication of malaria and agricultural land availability (pull) in the lowlands [63]. While the global population growth rate has declined since the late 1960s, substantial population increases are still expected in some regions, e.g. in sub-Saharan Africa, and are projected to be concentrated in low-income urban communities [64].

Households’ livelihood strategies, defined as income generating activities conditioned by assets and mediated by institutional and social relations [65], result from production related decisions (that, amongst other things, are based on pre-existing characteristics such as land and labour availability). Changes in production systems can directly affect livelihood strategies (N7). For example, the Chinese government’s conservation and development policies have lead to a vast sedentarization movement of Tibetan households and connectedly to a shift from a livestock rearing based livelihood to an agricultural-based livelihood [66]. The production system structure can also directly influence the type and level of health threats faced locally (N5), e.g. conversion of forest to agricultural production in Northern Thailand led to a decline in malaria threat caused by the *Anopheles* mosquito, occupying forest habitats [67].

Policies and budgets also influence medicinal plant demand by defining national health policies and thereby local access to health care options (N3), e.g. through public funding of hospitals and clinics [68-70]. Lastly, policies and budgets directly impact on the types and levels of disease threats faced locally, regionally, and nationally (N4), e.g. through provision of safe drinking water and sanitation or maintenance of disaster management regimes (e.g. [71,72]).

#### Local level

Medicinal plants are supplied from wild and domesticated vegetation types that can be described by their size, species composition, and quality. The global number of medicinal plant species is estimated at almost 53,000 [73], corresponding to 10-18% of the world’s vascular plant species [74]. Except for the few hundred species in cultivation [73] these are all wild harvested and there is thus a close link between renewable natural resources (forests, meadows, etc.) and human health. However, it should be noted that domestication takes place along a gradient of increasing human energy input per plant and that low energy input supply mechanisms may be important, e.g. households may access weeds [75] or maintain supplies in house gardens [76]. As biodiversity is degraded [77] the opportunities of medicinal plant use for health care will change.

Resource management systems, ranging from open access to complete protection, influence the potential and actual supply of medicinal plants (L1). In the past decades, a growing realization of the inability of central authorities to monitor, let alone manage, distant natural resources has led to increasing decentralization in developing countries [78]. In consequence, open access regimes give way to collective action with higher potentials for sustainable management and stable supply of products such as medicinal plants [79,80]. A limitation on management is lack of basic information on frequency and growth of most medicinal plant species [81]. But even in the absence of such information local cultivation may arise when the supply of highly demanded medicinal plants is threatened [82,83].

Health care options cover access to public and private health care choices. Traditionally, public health care systems in most countries are singular [84], with some notable exceptions such as in China and India, promoting the use of allopathic medicine with no or little public support for traditional medicine in terms of research, medical insurance or other aspects. Increasingly, however, public health care systems are becoming more pluralistic, incorporating traditional medicine practices to supplement allopathic treatment and to more effectively reach the rural population, e.g. in treatment of HIV/AIDS in Africa (e.g. [85-87]). There are also examples of allopathic therapists in developed countries using traditional medicine (e.g. [88,89]). There are very few studies of how increasing pluralistic public health care impacts on the user demand for medicinal plants (L2). Increased budgets for pluralistic public health care in developed countries would probably increase consumption of medicinal plants, whereas in developing countries, where public health care systems often have limited reach, the impact may be less pronounced, in particular outside urban areas.

Users’ demand for medicinal plants is influenced by the type and level of health threats that they are facing (L3) [90]. Type refers to the category of threat (e.g. infectious diseases) and level to the intensity of the threat (e.g. epidemic). Where a choice between allopathic and traditional medicine exists, e.g. in developed countries and among the better-off in urban areas of developing countries, it appears that allopathic medicines are often recurred to in case of serious (often infectious) diseases, whereas traditional medicine is more often used to counter mild diseases (e.g. [34,91]). For epidemics such as HIV/AIDS traditional medicine is likely to be used as a supplement or a last resort only [92]. In a number of developing countries, low public health budgets and subsequent lack of access to allopathic medicine lead to recurrence to traditional medicines when dealing with epidemics (e.g. [93]), especially in rural areas. Furthermore, increased resistance to allopathic drugs means that traditional medicines are sought to treat diseases such as malaria [94]. The higher frequency of serious diseases and generally higher threat levels expected as a consequence of climate changes will likely mean increased consumption of both allopathic and traditional medicine. It should be noted that different communities may have different adaptation capacities, e.g. in relation to climate changes. Such differences in vulnerability are not well understood [7].

Urbanization is increasing particularly in the tropics [95]. Migration and urbanization have been shown to affect the type and level of threats around the world (L4). For example, urbanization in Africa has lead to a profound decrease in morbidity and mortality from malaria due to a detrimental effect of city environments on anopheline species’ diversity, numbers, survival rates and infection rates [96].

#### Household level

Medicinal plant consumption is determined by demand and available stocks (H5, H6). As most medicinal plant species are harvested in the wild, the extractivism cycle proposed by Homma [60] provides a useful starting point for analyzing medicinal plant production over time at the single species level. Generally, as medicinal plant demand is probably growing while the resource base is shrinking, it will be challenging to maintain consumption in the future. While there are good published species level treatments of plant uses and location-specific studies with detailed species level stock and flow information, i.e. studies estimating available harvestable amounts and actual levels of extraction in particular locations (e.g. [97,98]), there are no studies providing stock and flow information for a species across its distribution range.

User demand for medicinal plants, expressed by individual decisions on preventive and curative treatment resorts, are influenced by user characteristics (H2). In developed countries, the use of traditional medicine is positively correlated with income [36,91,99], it is mainly used to maintain health [36,100,101] and it is related to a positive comparison with allopathic therapists in terms of patient care [92] and effectiveness [91,92,102]. It appears that traditional medicine expense is an additional cost that does not substitute allopathic medicine costs [99,103]. The proportion of the population aged over 60 years in developed countries is predicted to increase from 19% to 32% by 2050 [104] and it appears reasonable to assume that this will lead to an increased demand for both allopathic and traditional medicines. In developing countries, traditional medicine is resorted to because it is the only option (e.g. [69,105]) or the preferred option, e.g. due to better patient care (e.g. [106,107]). It is increasingly documented that people in developing countries resort to parallel treatments with traditional and allopathic medicine and that the choice is pragmatic rather than cultural (e.g. [108-111]). The increased availability of allopathic medicine in developing countries will therefore likely decrease the use of traditional medicine, but not displace it.

Livelihood strategies of households can be affected by patterns of migration^b^ and urbanization (H4). For example, migration is often a way to increase or diversify income and/or to ensure access to assets for rural populations [112]. In turn, livelihood strategies influence household and individual medicinal plant user characteristics (H3), e.g. through the physical and human assets available for investing in disease preventive measures such as boiling drinking water [72] and financial assets available for meeting treatment costs.

User demand for medicinal plants is thus shaped by user characteristics (H2), and determined by available health care options (L3) and perceptions of threats (L4), as well as by medicinal plant supply (H1). Again, note the dual nature of the directional causality, e.g. user preferences can both promote and discourage the consumption of medicinal plants.

## Discussion

Medicinal plants appear to particularly provide poorer people in developing countries with affordable health care options, and well-off people in developed countries with health maintenance options. Current general development trends in developing (population increase, poor coverage of western health care, accessibility of traditional medicines) and developed (aging populations) countries indicate that medicinal plant consumption is not likely to decrease in the short to medium term - consumer, producer and society-wide medicinal plant benefits will persist. Therefore, and regardless of the constraints to the development of a sound evidence base on safety and efficacy for herbal medicines [113] and related products, we should improve our understanding of what drives medicinal plant consumption. Information on these drivers will constitute important building blocks in designing pluralistic health policies and improved natural resources management interventions to the benefits of hunter-gatherers, farmers and pastoralists, urban and peri-urban people, and entrepreneurs across the globe.

The presented unified conceptual framework offers a first step towards establishing a comprehensive approach to understanding the dynamics of medicinal plant consumption. At present the literature is dominated by studies that are disciplinary or sectoral focused; while many of these are high quality and informative in themselves, the lack of a general conceptual framework makes it difficult to pinpoint what spatial levels and causal factors need attention. The framework presented here fills in this knowledge gap by providing a structured approach to systematically investigate changes in medicinal plant consumption based on changes in key factors. For instance, it emphasizes the importance of assessing the impact caused by changes in land use patterns on medicinal plant consumption. An alternative approach to the presented framework would be to develop a mathematical model that would allow more detailed analysis, e.g. of feed-back loops. However, given existing data gaps and the lack of knowledge on key factors, such models would require a heroic number of assumptions and would be less transparent than the proposed framework.

At present, a systematic endeavour to fill the vast knowledge gaps in our understanding of medicinal plant consumption dynamics is needed to inform future health and natural resource management policies. Apart from increasing our conceptual understanding of medicinal plant consumption dynamics, the proposed framework can serve to guide research towards systematically pursuing this objective. Standardized international or national surveys do not presently include the concept of medicinal plant reliance, and in many cases only limited information on the main causal factors and linkages to medicinal plant consumption will be available at the country level, thus making it difficult to measure the central variables (assessing the strength of factors, linkages and their impacts on future consumption). Therefore, a next step in operationalising the framework could be to develop an analytical framework enabling country-level comparative studies of changes in medicinal plant consumption, e.g. through the identification of a set of generic indicators and a research protocol.

### Limitations of the framework

We acknowledge that creating a single unified framework aimed at uncovering the determinants affecting medicinal plant consumption at the international, national, local and household levels is a bold venture; however, this comprehensive approach is necessary to illustrate and understand the multiple and complex factors influencing medicinal plant consumption. A similar approach has been successfully used to create a framework that formed the basis for analytical dissection and understanding of the complexities of tropical deforestation [114].

It should be noted that the presented conceptual framework does not portray the full complexity of linkages, nor does it depict temporal (bi-directional) linkages, e.g. resource management systems will over time impact on medicinal plant resource productivity. It should also be noted that the factors and linkages constitute a “gross list” of what is potentially important – not all factors and linkages will be important in any particular geographical location and their relative importance may vary across time.

The four medicinal plant user groups depicted in the first part of the paper are based on generalizations (e.g. hunter-gatherer communities are generally seen as remote and poor while farmers are generally seen as wealthier and with access to better infrastructure). While acknowledging the risk of excluding certain communities which do not conform to the general patterns observed from the literature, we consider the four groups a useful structure when reflecting on which types of people and communities are reliant on medicinal plants.

## Conclusions

Current evidence indicates that a huge number of people rely on medicinal plant products to maintain their health or treat illnesses, and that this number is unlikely to decrease in the foreseeable future. The paper inductively synthesises available scattered knowledge on medicinal plant production, trade and consumption to propose a conceptual framework identifying the factors, and their interconnectedness, determining medicinal plant consumption. The framework is based on a typology of main medicinal plant user groups (hunter-gatherers, farmers and pastoralists, urban and peri-urban residents, and entrepreneurs) and three basic kinds of benefits (producer, consumer, and society-wide). Factors and linkages in the proposed framework range from international to household levels and, though necessarily broad, it can thus facilitate the construction of internationally comparable knowledge. The proof of success, however, is whether the proposed framework will stimulate research that is empirically and theoretically richer than in the past and whether the resultant outcomes will more effectively contribute to improved human health and better medicinal plant resource management.

## End notes

^a^ Although it has been argued that the term traditional medicine should only be used for medicine which has been commonly practiced for more than a generation [115,116], we adopt a WHO working definition of traditional medicine: “Health practices, approaches, knowledge and beliefs incorporating plant, animal and mineral based medicines, spiritual therapies, manual techniques and exercises, applied singularly or in combination to treat, diagnose and prevent illnesses or maintain well-being” [117]. A crude distinction can be made between ‘imported’ and ‘native’ traditional medicines. For instance, some traditional medicine practices have been imported to Europe with migrants (e.g. [118]) and could be termed ‘imported’; however, the distinction becomes blurred over time. Far from all components of traditional medicine include use of medicinal plants. Traditional and allopathic medicine systems may occur side by side in the same location. Allopathic medicine is the term used for industrially produced pure, standardized compounds, which are tested for efficacy and side-effects.

^b^ In analyzing migration, human patterns of spatial mobility, it is useful to distinguish international and domestic migration, and permanent and short-term migration. The latter is also known as circulation. There is no single generally accepted model of migration [64] and projections are difficult. In the framework, the “Migration and urbanization” factor includes all kinds of migration (the relevant kind of migration will vary with the case being studied).

## Competing interests

The authors declare that they have no competing interests.

## Authors’ contributions

The paper is part of the work of the medicinal plant group at theDepartment. It was conceived by CSH and HOL. HOL undertook database searches. Text in all sections and the framework was developed jointly by all authors. All authors read and approved the final manuscript.

## Supplementary Material

Additional file 1 Appendix. Click here for file
