# Supplementary material for: People, plants and health: a conceptual framework for assessing changes in medicinal plant consumption
Source: J Ethnobiol Ethnomed. 2012 Nov 13;8:43. doi: 10.1186/1746-4269-8-43 (PMC3549945; doi:10.1186/1746-4269-8-43)
Supplement: Additional file 1 — Appendix. [file 1746-4269-8-43-S1.rtf]

Appendix
This Appendix contains full references for the sources located as specified in the Methods section. Included at the end of each reference is a specification of the relevant linkage(s) in the conceptual framework. In total are specified 931 linkages (higher than the number of references as some sources cover more than one linkage). Local and household levels are generally well-covered while linkages at international and national levels have received much less attention. The 931 linkages are distributed (link no./no. of references): I1/2, I2/3, I3/2, I4/5, N1/8, N2/4, N3/57, N4/2, N5/1, N6/3, N7/1, L1/58, L2/158, L3/256, L4/8, H1/10, H2/292, H3/26, H4/30, H5/2  and H6/2.

Aarons DE: Medicine and its alternatives. Health care priorities in the Caribbean. Hastings Cent Rep 1999, 29: 23-27. N3.
Ab RR: Knowledge, attitude and practice of Malay folk methods in family planning. Malays J Reprod Health 1985, 3: S64-S81. L3, H3, H4.
Abadoglu O, Cakmak E, Kuzucu DS: The view of patients with asthma or chronic obstructive pulmonary disease (COPD) on complementary and alternative medicine. Allergol Immunopathol (Madr ) 2008, 36: 21-25. L3.
Abdullah Al-Rowais N, Al Bedah AM, Khalil MK, El Olemy AT, Khalil AA, Alrasheid MH et al.: Knowledge and attitudes of primary health care physicians towards complementary and alternative medicine in the riyadh region, saudi arabia. Forsch Komplementmed 2012, 19: 7-12. L2.
Abu-Qamar MZ, Wilson A: Qualitative Content Analysis of Complementary Topical Therapies Used to Manage Diabetic Foot in Jordan. African Journal of Traditional Complementary and Alternative Medicines 2012, 9: 440-448. L3.
Aceves-Avila FJ, Medina F, Fraga A: Herbal therapies in rheumatology: the persistence of ancient medical practices. Clin Exp Rheumatol 2001, 19: 177-183. L3.
Adams J, Sibbritt D, Young AF: Naturopathy/herbalism consultations by mid-aged Australian women who have cancer. European Journal of Cancer Care 2005, 14: 443-447. L3.
Adejuyigbe EA, Odebiyi AI, Aina O, Bamiwuye S: Feeding and care of low-birthweight babies in two rural communities in south-western Nigeria. Matern Child Nutr 2008, 4: 55-64. L3, H3.
Adekolu-John EO: Maternal health care and outcome of pregnancies in Kainji Lake Area of Nigeria. Public Health 1989, 103: 41-49. L3, L2.
Adera TD: Beliefs and traditional treatment of malaria in Kishe settlement area, southwest Ethiopia. Ethiopian Medical Journal 2003, 41:25-34. H2.
Adnan M, Hofscher D: Medicinal Plant Abundance in Degraded and Reforested Sites in Northwest Pakistan. Mountain Research and Development 2010, 30: 25-32. L1.
Adusi-Poku Y, Okine LK, Hlortsi-Akakpo FK, Fleischer TC, Mensah ML, Arhin P et al.: Assesssing herbal medical practitioners in professional qualifying examination in Ghana, a model. Afr J Tradit Complement Altern Med 2010, 7: 85-87. L2.
Afifi FU, Wazaify M, Jabr M, Treish E: The use of herbal preparations as complementary and alternative medicine (CAM) in a sample of patients with cancer in Jordan. Complement Ther Clin Pract 2010, 16: 208-212. L3.
Agadjanian V: Women's choice between indigenous and Western contraception in urban Mozambique. Women Health 1998, 28: 1-17. L3.
Agbor AM, Naidoo S: Knowledge and practice of traditional healers in oral health in the Bui Division, Cameroon. J Ethnobiol Ethnomed 2011, 7: 6. L2, L3.
Aginam O: Beyond shamanism: the relevance of African traditional medicine in global health policy. Med Law 2007, 26: 191-201. N3.
AhChing LP, Sapolu M, Samifua M, Yamada S: Attitudes regarding tuberculosis among Samoans. Pac Health Dialog 2001, 8: 15-19. L3.
Ahmad SS, Javed S: Exploring the economic value of 1435 underutilized plant species in Ayubia National Park. Pakistan Journal of Botany 2007, 39: 1435-1442. H1.
Ahmad SS: Medicinal wild plants from Lahore-Islamabad motorway (M-2). Pakistan Journal of Botany 2007, 39: 355-375. H1.
Ahmad SS, Husain SZ: Ethno medicinal survey of plants from salt range (Kallar Kahar) of Pakistan. Pakistan Journal of Botany 2008, 40: 1005-1011. H2.
Ahorlu CK, Dunyo SK, Koram KA, Nkrumah FK, Aagaard-Hansen J, Simonsen PE: Lymphatic filariasis related perceptions and practices on the coast of Ghana: implications for prevention and control. Acta Trop 1999, 73: 251-261. L2.
Aikins MK, Pickering H, Greenwood BM: Attitudes to malaria, traditional practices and bednets (mosquito nets) as vector control measures: a comparative study in five west African countries. J Trop Med Hyg 1994, 97: 81-86. L3.
Ailinger RL, Molloy S, Zamora L, Benavides C: Herbal remedies in a Nicaraguan barrio. J Transcult Nurs 2004, 15: 278-282. L2.
Ajaiyeoba EO, Oladepo O, Fawole OI, Bolaji OM, Akinboye DO, Ogundahunsi OA et al.: Cultural categorization of febrile illnesses in correlation with herbal remedies used for treatment in Southwestern Nigeria. J Ethnopharmacol 2003, 85: 179-185. H2, H3, H4.
Ajewole JF, Faal HB, Johnson G, Hart A: Understanding the community perspectives of trachoma: The Gambia as a case study. Ophthalmic Epidemiol 2001, 8: 163-180. L3.
Akan H, Rak G, Kaspar EC, CD N, Ayd 305 N S, Demircan N et al.: Knowledge and attitudes towards complementary and alternative medicine among medical students in Turkey. BMC Complement Altern Med 2012, 12: 115. L2.
Akerreta S, Cavero RY, Lopez V, Calvo MI: Analyzing factors that influence the folk use and phytonomy of 18 medicinal plants in Navarra. J Ethnobiol Ethnomed 2007, 3: 16. H1, H2.
Akogun OB, John KK: Illness-related practices for the management of childhood malaria among the Bwatiye people of north-eastern Nigeria. Malar J 2005, 4: 13. L3.
Akyol AD, Oz B: The use of complementary and alternative medicine by patients with cancer: in Turkey. Complement Ther Clin Pract 2011, 17: 230-234. L3.
Al-Jaroudi DH: Beliefs of subfertile Saudi women. Saudi Med J 2010, 31: 425-427. L3.
Alkhuzai J, Al-Laith A, Mandeel Q: Knowledge, attitudes, and practices regarding medicinal plants used by the indigenous people in Bahrain. Arab Gulf Journal of Scientific Research 2010, 28:105-117. H2, L3.
Al-Rowais NA, Al Bedah AMN, Khalil MKM, El Olemy AT, Khalil AAH, Alrasheid MHS et al.: Knowledge and Attitudes of Primary Health Care Physicians Towards Complementary and Alternative Medicine in the Riyadh Region, Saudi Arabia. Forschende Komplementarmedizin 2012, 19: 7-12. L2.
Al-Saeedi M, Elzubier AG, Bahnassi AA, Al-Dawood KM: Patterns of belief and use of traditional remedies by diabetic patients in Mecca, Saudi Arabia. East Mediterr Health J 2003, 9: 99-107. L3.
AlBraik FA, Rutter PM, Brown D: A cross-sectional survey of herbal remedy taking by United Arab Emirate (UAE) citizens in Abu Dhabi. Pharmacoepidemiology and Drug Safety  2008, 17: 725-732. H2.
Alencar NL, Araujo TAD, de Amorim ELC, de Albuquerque UP: Can the Apparency Hypothesis explain the selection of medicinal plants in an area of caatinga vegetation? A chemical perspective. Acta Botanica Brasilica 2009, 23: 910-911. L2.
Algier LA, Hanoglu Z, Ozden G, Kara F: The use of complementary and alternative (non-conventional) medicine in cancer patients in Turkey. Eur J Oncol Nurs 2005, 9: 138-146. L3. 
Ali-Shtayeh MS, Jamous RM, Jamous RM: Herbal preparation use by patients suffering from cancer in Palestine. Complement Ther Clin Pract 2011, 17: 235-240. H2.
Ali AM: [The utilization of nurses and community public health workers in the rural areas of developing countries]. Med Sestra 1989, 48: 19-22. L2.
Alkhuzai J, Mandeel Q, Al-Laith A: Folk Medicine and the Use of Herbal Plants in the Kingdom of Bahrain. Arab Gulf Journal of Scientific Research 2010, 28: 91-104. H2.
Alkhuzai J, Al-Laith A, Mandeel Q: Knowledge, Attitudes, and Practices regarding Medicinal Plants Used by the Indigenous People in Bahrain. Arab Gulf Journal of Scientific Research 2010, 28: 105-117. H2.
Allali H, Benmehdi H, Dib MA, Tabti B, Ghalem S, Benabadji N: Phytotherapy of diabetes in West Algeria. Asian Journal of Chemistry 2008, 20: 2701-2710. L3.
Allen R, Cushman LF, Morris S, Feldman J, Wade C, McMahon D et al.: Use of complementary and alternative medicine among Dominican emergency department patients. Am J Emerg Med 2000, 18: 51-54. H2.
Almas K, Albaker A, Felembam N: Knowledge of dental health and diseases among dental patients, a multicentre study in Saudi Arabia. Indian J Dent Res 2000,  11: 145-155. L3.
Altan S: The Herbalists: The Use of Medicinal Plants and Ethical Problems. Turkiye Klinikleri Tip Bilimleri Dergisi 2008, 28: S209-S212. N3.
Anderson FW, Naik SI, Feresu SA, Gebrian B, Karki M, Harlow SD: Perceptions of pregnancy complications in Haiti. Int J Gynaecol Obstet 2008, 100: 116-123. L3.
Anokbonggo WW, Odoi-Adome R, Oluju PM: Traditional methods in management of diarrhoeal diseases in Uganda. Bull World Health Organ 1990, 68: 359-363. L2.
	Anonymous: Self-treatment with herbal and other plant-derived remedies--rural Mississippi, 1993. MMWR Morb Mortal Wkly Rep 1995, 44: 204-207. H2.
Anonymous: TBAs drive project in Guatemala. JOICFP News 1996, 6. L2.
Anonymous: Tribal voices fight for equity. Health Millions 1999, 25: 8-9. L2.
Anonymous: Alternative medicine, DMPA costs top concerns. Contracept Technol Update 1999, 20: 110-111. L2, H2.
Anonymous: San Francisco: HMO coverage of acupuncture. AIDS Treat News 1999, 8. L3.
Anonymous: [WHO guidelines for governments and consumers regarding the use of alternative therapies]. Rev Panam Salud Publica 2004, 16: 218-221. I4, N3.
Anyinam C: Availability, accessibility, acceptability, and adaptability: four attributes of African ethno-medicine. Soc Sci Med 1987, 25: 803-811. H2.
Anyinam C: Ecology and ethnomedicine: exploring links between current environmental crisis and indigenous medical practices. Soc Sci Med 1995, 40: 321-329. L1.
Anyonge CH, Rugalema G, Kayambazinthu D, Sitoe A, Barany M: Fuelwood, food and medicine: the role of forests in the response to HIV and AIDS in rural areas of southern Africa. Unasylva 2006, 57:20-23. L1.
Applewhite SL: Curanderismo: demystifying the health beliefs and practices of elderly Mexican Americans. Health Soc Work 1995, 20: 247-253. H2.
Arkovitz MS, Manley M: Specialization and referral among the n'anga (traditional healers) of Zimbabwe. Trop Doct 1990, 20: 109-110. L2.
Armitage GD, Suter E, Verhoef MJ, Bockmuehl C, Bobey M: Women's needs for CAM information to manage menopausal symptoms. Climacteric 2007, 10: 215-224. L3.
Armstrong AR, Thiebaut SP, Brown LJ, Nepal B: Australian adults use complementary and alternative medicine in the treatment of chronic illness: a national study. Aust N Z J Public Health 2011, 35: 384-390. L3.
Armstrong TS, Gilbert MR: Use of complementary and alternative medical therapy by patients with primary brain tumors. Curr Neurol Neurosci Rep 2008, 8: 264-268. L3.
Ashikaga T, Bosompra K, O'Brien P, Nelson L: Use of complimentary and alternative medicine by breast cancer patients: prevalence, patterns and communication with physicians. Support Care Cancer 2002, 10: 542-548. L3.
Ashworth TG: Family demography in a remote rural community in Zambia. Public Health 1998, 112: 313-316. H2.
Astin JA: Why patients use alternative medicine: results of a national study. Journal of the American Medical Association 1998, 279:1548-1553. H2.
Aubel J, Rabei H, Mukhtar M: Health workers' attitudes can create communication barriers. World Health Forum 1991, 12: 466-471. L2.
Ayisi JG, van't Hoog AH, Agaya JA, Mchembere W, Nyamthimba PO, Muhenje O et al.: Care seeking and attitudes towards treatment compliance by newly enrolled tuberculosis patients in the district treatment programme in rural western Kenya: a qualitative study. BMC Public Health 2011, 11: 515. L3.
Azaizeh H, Fulder S, Khalil K, Said O: Ethnobotanical knowledge of local Arab practitioners in the Middle Eastern region. Fitoterapia 2003, 74: 98-108. L2.
Aziz Z, Tey NP: Herbal medicines: prevalence and predictors of use among Malaysian adults. Complement Ther Med 2009, 17: 44-50. H2.
Baguma P: The traditional treatment of AIDS in Uganda: benefits and problems. Key issues and debates: traditional healers. Soc Afr SIDA 1996, 4-6. L3.
Bair YA, Gold EB, Greendale GA, Sternfeld B, Adler SR, Azari R et al.: Ethnic differences in use of complementary and alternative medicine at midlife: longitudinal results from SWAN participants. Am J Public Health 2002, 92: 1832-1840. H2.
Balick MJ, Kronenberg F, Ososki AL, Reiff M, Fugh-Berman A, O'Connor B et al.: Medicinal plants used by Latino healers for women's health conditions in New York City. Economic Botany 2000, 54: 344-357. H2, H3, H4.
Balick MJ, Lee R: Looking within: urban ethnomedicine and ethnobotany. Altern Ther Health Med 2001, 7: 114-115. H2, H3, H4.
Balick MJ, De Gezelle JM, Arvigo R: Feeling the pulse in Maya medicine: an endangered traditional tool for diagnosis, therapy, and tracking patients' progress. Explore (NY) 2008, 4: 113-119. L2.
Balslev H: Palm Harvest Impacts in North-Western South America. Botanical Review 2011, 77: 370-380. L1.
Baltussen R, Ye Y: Quality of care of modern health services as perceived by users and non-users in Burkina Faso. International Journal for Quality in Health Care 2006, 18:30-34. L2.
Bamidele JO, Adebimpe WO, Oladele EA: Knowledge, attitude and use of alternative medical therapy amongst urban residents of Osun State, southwestern Nigeria. Afr J Tradit Complement Altern Med 2009, 6: 281-288. H2, H3, H4.
Bannerman RH: Traditional medicine in modern health care. World Health Forum 1982, 3:8-13. L2.
Bardazzi G, Merluzzi JA, Voller F, Fontana A, Abenavoli L, Leggio L et al.: Complementary medicine for alcohol dependence in Italian services: a mail questionnaire. Complement Ther Clin Pract 2006, 12: 216-221. L3.
Baron SE, Goodwin RG, Nicolau N, Blackford S, Goulden V: Use of complementary medicine among outpatients with dermatologic conditions within Yorkshire and South Wales, United Kingdom. J Am Acad Dermatol 2005, 52: 589-594. H2.
Barreto A: [The secret of healers (Brazil)]. Med Trop (Mars ) 1991, 51: 421-427. L2.
Barrett B: Ethnomedical interactions: health and identity on Nicaragua's Atlantic coast. Soc Sci Med 1995, 40: 1611-1621. N3.
Barrett B: Complementary and alternative medicine: what's it all about? WMJ 2001, 100: 20-26. H2.
Barrett B: Alternative, complementary, and conventional medicine: is integration upon us? J Altern Complement Med 2003, 9: 417-427. N3.
Barrett B, Marchand L, Scheder J, Plane MB, Maberry R, Appelbaum D et al.: Themes of holism, empowerment, access, and legitimacy define complementary, alternative, and integrative medicine in relation to conventional biomedicine. Journal of Alternative and Complementary Medicine 2003, 9: 937-947. L2.
Barron F, Hunter A, Mayo R, Willoughby D: Acculturation and adherence: issues for health care providers working with clients of Mexican origin. J Transcult Nurs 2004, 15: 331-337. L2.
Barry JA: Living with type 2 diabetes mellitus in a modern Mexican city and opting for phytotherapy. Explore (NY) 2012, 8: 48-49. L3.
Bastien JW: Herbal curing by Qollahuaya andeans. J Ethnopharmacol 1982, 6: 13-28. H4.
Bastien JW: Exchange between Andean and Western medicine. Soc Sci Med 1982, 16: 795-803. L2.
Beebe-Dimmer JL, Wood DP, Jr., Gruber SB, Douglas JA, Bonner JD, Mohai C et al.: Use of complementary and alternative medicine in men with family history of prostate cancer: a pilot study. Urology 2004, 63: 282-287. L3.
Bekalo TH, Woodmatas SD, Woldemariam ZA: An ethnobotanical study of medicinal plants used by local people in the lowlands of Konta Special Woreda, southern nations, nationalities and peoples regional state, Ethiopia. J Ethnobiol Ethnomed 2009, 5: 26. L1.
Bello FA, Morhason-Bello IO, Olayemi O, Adekunle AO: Patterns and predictors of self-medication amongst antenatal clients in Ibadan, Nigeria. Niger Med J 2011, 52: 153-157. L3.
Belton S, Whittaker A: Kathy Pan, sticks and pummelling: techniques used to induce abortion by Burmese women on the Thai border. Soc Sci Med 2007, 65: 1512-1523. H2.
Ben-Arye E, Shapira C, Keshet Y, Hogerat I, Karkabi K: Attitudes of Arab-Muslims toward integration of complementary medicine in primary-care clinics in Israel: the Bedouin mystery. Ethn Health 2009, 14: 379-391. H2.
Ben-Arye E, Lev E, Keshet Y, Schiff E: Integration of herbal medicine in primary care in Israel: a jewish-arab cross-cultural perspective. Evid Based Complement Alternat Med 2011, 2011: 401395. H2.
Benzi G, Ceci A: Herbal medicines in European regulation. Pharmacol Res 1997, 35: 355-362. N3.
Berenzon S, Saavedra N: Present of the herbal medicine in the treatment of emotional problems: narrations of urban healers. Salud Mental 2002, 25: 55-66. H2, L3.
Bernstein BJ, Grasso T: Prevalence of complementary and alternative medicine use in cancer patients. Oncology (Williston Park) 2001, 15: 1267-1272. L3.
Bertomoro P, Renna S, Cottone M, Riegler G, Bossa F, Giglio L et al.: Regional variations in the use of complementary and alternative medicines (CAM) for inflammatory bowel disease patients in Italy: an IG-IBD study. J Crohns Colitis 2010, 4: 291-300. L3.
Bhasin V: Social Change, Religion and Medicine among Brokpas of Ladakh. Studies on Ethno-Medicine 2008, 2: 77-102. H2.
Bhopal RS: The inter-relationship of folk, traditional and Western medicine within an Asian community in Britain. Soc Sci Med 1986, 22: 99-105. L2, H2.
Bibeau G: New legal rules for an old art of healing: the case of Zairian Healers' Association. Soc Sci Med 1982, 16: 1843-1849. N3.
Bica I, Tang AM, Skinner S, Spiegelman D, Knox T, Gorbach S et al.: Use of complementary and alternative therapies by patients with human immunodeficiency virus disease in the era of highly active antiretroviral therapy. J Altern Complement Med 2003, 9: 65-76. L3.
Bielory L: Complementary medicine for the allergist. Allergy Asthma Proc 2001, 22: 33-37. N3.
Bierlich B: Sacrifice, plants, and western pharmaceuticals: money and health care in northern Ghana. Med Anthropol Q 1999, 13: 316-337. H2.
Birhan W, Giday M, Teklehaymanot, T: The contribution of traditional healers' clinics to public health care system in Addis Ababa, Ethiopia: a cross-sectional study. Journal of Ethnobiology and Ethnomedicine 2011, 7:39. L2.
Bishop FL, Prescott P, Chan YK, Saville J, von EE, Lewith GT: Prevalence of complementary medicine use in pediatric cancer: a systematic review. Pediatrics 2010, 125: 768-776. H2, L3.
Bjersa K, Victorin ES, Olsen MF: Knowledge about complementary, alternative and integrative medicine (CAM) among registered health care providers in Swedish surgical care: a national survey among university hospitals. Bmc Complementary and Alternative Medicine 2012, 12. L2.
Bjornsdottir I, Almarsdottir AB, Traulsen JM: The lay public's explicit and implicit definitions of drugs. Res Social Adm Pharm 2009, 5: 40-50. H2.
Blalock SJ, Gregory PJ, Patel RA, Norton LL, Callahan LF, Jordan JM: Factors associated with potential medication-herb/natural product interactions in a rural community. Altern Ther Health Med 2009, 15: 26-34. H2.
Blanchard DS, Bean A: Healing practices of the people of Belize. Holist Nurs Pract 2001, 15: 70-78. L2.
Bland RM, Rollins NC, Van den Broeck J, Coovadia HM: The use of non-prescribed medication in the first 3 months of life in rural South Africa. Trop Med Int Health 2004, 9: 118-124. L3.
Bliwise DL, Ansari FP: Insomnia associated with valerian and melatonin usage in the 2002 National Health Interview Survey. Sleep 2007, 30: 881-884. H2.
Blum LS, Oria PA, Olson CK, Breiman RF, Ram PK: Examining the use of oral rehydration salts and other oral rehydration therapy for childhood diarrhea in Kenya. Am J Trop Med Hyg 2011, 85: 1126-1133. H3.
Bodeker G: Traditional medical knowledge, intellectual property rights and benefit sharing. Cardozo Journal of International and Comparative Law 2001, 11:785-814. I4.
Bodeker G, Carter G, Burford G, Dvorak-Little M: HIV/AIDS: Traditional systems of health care in the management of a global epidemic. J Altern Complement Med 2006, 12: 563-576. L3.
Bodenstein JW: Observations on medicinal plants. S Afr Med J 1973, 47: 336-338. H2.
Booker A, Johnston D, Heinrich M: Value chains of herbal medicines--research needs and key challenges in the context of ethnopharmacology. J Ethnopharmacol 2012, 140: 624-633. H3.
Boon H, Stewart M, Kennard MA, Gray R, Sawka C, Brown JB et al.: Use of complementary/alternative medicine by breast cancer survivors in Ontario: prevalence and perceptions. J Clin Oncol 2000, 18: 2515-2521. L3.
Boon H: Regulation of complementary/alternative medicine: a Canadian perspective. Complement Ther Med 2002, 10: 14-19. N3.
Boon HS, Cherkin DC, Erro J, Sherman KJ, Milliman B, Booker J et al.: Practice patterns of naturopathic physicians: results from a random survey of licensed practitioners in two US States. BMC Complement Altern Med 2004, 4: 14. L2, L3.
Breitbart V, Morales H, Brown J, Betances B, Kahalnik F: Con un pie en dos islas: cultural bridges that inform sexual and reproductive health in the Dominican Republic and New York. Cult Health Sex 2010, 12: 543-554. H2, H3, H4.
Breuner CC: Alternative and complementary therapies. Adolesc Med Clin 2006, 17: 521-546. L2.
Bridevaux IP: A survey of patients' out-of-pocket payments for complementary and alternative medicine therapies.  Complement Ther Med 2004, 12: 48-50. H2.
Broome B, Broome R: Native Americans: traditional healing. Urol Nurs 2007, 27: 161-3, 173. L2.
Brown CM, Pena A, Resendiz K: Pharmacists' actions when patients use complementary and alternative medicine with medications: A look at Texas-Mexico border cities. J Am Pharm Assoc (2003 ) 2011, 51: 619-622. L2.
Brunelli B, Gorson KC: The use of complementary and alternative medicines by patients with peripheral neuropathy. J Neurol Sci 2004, 218: 59-66. L3.
Bruni A, Ballero M, Poli F: Quantitative ethnopharmacological study of the Campidano valley and Urzulei district, Sardinia, Italy. Journal of Ethnopharmacology 1997, 57: 97-124. H4.
Bucker B, Groenewold M, Schoefer Y, Schafer T: The use of complementary alternative medicine (CAM) in 1 001 German adults: results of a population-based telephone survey. Gesundheitswesen 2008, 70: e29-e36. H2.
Buehler BA: Complementary and alternative medicine (CAM) in genetics. Am J Med Genet A 2007, 143A: 2889-2892. H2.
Burg MA, Hatch RL, Neims AH: Lifetime use of alternative therapy: a study of Florida residents. South Med J 1998, 91: 1126-1131. H2.
Busia K: Medical provision in Africa -- past and present. Phytother Res 2005, 19: 919-923. H2.
Bussing A, Ostermann T, Heusser P, Matthiessen PF: Usage of alternative medical systems, acupuncture, homeopathy and anthroposophic medicine, by older German adults. Zhong Xi Yi Jie He Xue Bao 2011, 9: 847-856. H2.
Bussmann RW, Sharon D: Markets, healers, vendors, collectors: the sustainability of medicinal plant use in northern Peru. Mountain Research and Development 2009, 29:128-134. H3.
Bussmann RW, Sharon D, Vandebroek I, Jones A, Revene Z: Health for sale: the medicinal plant markets in Trujillo and Chiclayo, Northern Peru. J Ethnobiol Ethnomed 2007, 3: 37. L1.
Bussmann RW, Swartzinsky P, Worede A, Evangelista P: Plant use in Odo-Bulu and Demaro, Bale region, Ethiopia. J Ethnobiol Ethnomed 2011, 7: 28. H2.
Cameron MM: Modern desires, knowledge control, and physician resistance: regulating Ayurvedic medicine in Nepal. Asian Medicine 2008, 4:86-112. N3.
Campbell DG, Turner AP, Williams RM, Hatzakis M, Bowen JD, Rodriquez A et al.: Complementary and alternative medicine use in veterans with multiple sclerosis: Prevalence and demographic associations. Journal of Rehabilitation Research and Development 2006, 43: 99-109. L3,
Campbell S: Traditional medicine in The Gambia. Complement Ther Nurs Midwifery 1997, 3: 103-105. L2.
Campos-Navarro R, Torrez D, Arganis-Juarez EN: [Representations of illness among the elderly in Mexico City].  Cad Saude Publica 2002, 18: 1271-1279. H2.
Canter PH, Thomas H, Ernst E: Bringing medicinal plants into cultivation: opportunities and challenges for biotechnology. Trends in Biotechnology 2005, 23:180-185. L1.
Cardini F, Lesi G, Lombardo F, van der Sluijs C: The use of Complementary and Alternative Medicine by women experiencing menopausal symptoms in Bologna. BMC Womens Health 2010, 10: 7. L3.
Carlton AL, Bensoussan A: Regulation of complementary medicine practitioners in Australia: Chinese medicine as a case example. Complement Ther Med 2002, 10: 20-26. N3.
Carne X: [The new European regulation on herbal traditional drugs. Application to the Spanish regulation]. Med Clin (Barc ) 2003, 121: 655-657. N3.
Carod FJ, Vazquez-Cabrera C: [Magical thinking and epilepsy in traditional indigenous medicine]. Rev Neurol 1998, 26: 1064-1068. L3.
Carrubba A, Scalenghe R: The scent of Mare Nostrum: medicinal and aromatic plants in Mediterranean soils. J Sci Food Agric 2012, 92: 1150-1170. L1.
Carvalho ACB, dos Santos LA, Silveira D: Regulation of plants and herbal medicines in Brazil. Boletin Latinoamericano y del Caribe de Plantas Medicinales y Aromaticas 2009, 8: 7-11. N3.
Cavender AP, Beck SH: Generational Change, Folk Medicine, and Medical Self-Care in A Rural Appalachian Community. Human Organization 1995, 54: 129-142. H2.
Ceuterick M, Vandebroek I, Torry B, Pieroni A: Cross-cultural adaptation in urban ethnobotany: the Colombian folk pharmacopoeia in London. J Ethnopharmacol 2008, 120: 342-359. H2, H3, H4.
Ceuterick M, Vandebroek I, Pieroni A: Resilience of Andean urban ethnobotanies: a comparison of medicinal plant use among Bolivian and Peruvian migrants in the United Kingdom and in their countries of origin. J Ethnopharmacol 2011, 136: 27-54. H2, H3, H4.
Chacko E: Culture and therapy: complementary strategies for the treatment of type-2 diabetes in an urban setting in Kerala, India. Soc Sci Med 2003, 56: 1087-1098. H2.
Challand S: A survey of the use of medicinal plants and other traditional medicine in Kasese District, Uganda. Trop Doct 2005, 35: 222-224. H2.
Chambers S, Raine R, Rahman A, Hagley K, De CK, Isenberg D: Factors influencing adherence to medications in a group of patients with systemic lupus erythematosus in Jamaica. Lupus 2008, 17: 761-769. L3.
Chang HK: Hawaiian health practitioners in contemporary society. Pac Health Dialog 2001, 8: 260-273. L2.
Chang LC, Huang N, Chou YJ, Lee CH, Kao FY, Huang YT: Utilization patterns of Chinese medicine and Western medicine under the National Health Insurance Program in Taiwan, a population-based study from 1997 to 2003. BMC Health Serv Res 2008, 8: 170. H2.
Chang LC, Huang N, Chou YJ, Kao FY, Hsieh PC, Huang YT: Patterns of combined prescriptions of aspirin-Ginkgo biloba in Taiwan: a population-based study. J Clin Pharm Ther 2008, 33: 243-249. L2.
Chang LH, Wang J: Integration of complementary medical treatments with rehabilitation from the perspectives of patients and their caregivers: a qualitative inquiry. Clin Rehabil 2009, 23: 730-740. L2, H2.
Chen FP, Chen TJ, Kung YY, Chen YC, Chou LF, Chen FJ et al.: Use frequency of traditional Chinese medicine in Taiwan. BMC Health Serv Res 2007, 7: 26. H2.
Chen H, Tu SP, Teh CZ, Yip MP, Choe JH, Hislop TG et al.: Lay beliefs about hepatitis among North American Chinese: implications for hepatitis prevention. J Community Health 2006, 31: 94-112. L3.
Chen S, Yin D, Li L, Zha X, Shuen J, Zhama C: [Resources and distribution of Cordyceps sinensis in Naqu Tibet]. Zhong Yao Cai 2000, 23: 673-675. L1.
Chen SL, Zhou YQ, Xie CX, Zhao RH, Sun CZ, Wei JH et al.: [Suitability evaluation of Panax quinquefolium's producing area based on TC. Zhongguo Zhong Yao Za Zhi 2008, 33: 741-745. L1.
Chen YC, Chen FP, Chen TJ, Chou LF, Hwang SJ: Patterns of traditional Chinese medicine use in patients with inflammatory bowel disease: a population study in Taiwan. Hepatogastroenterology 2008, 55: 467-470. L3.
Cheon-Klessig Y, Camilleri DD, Mc Elmurry BJ, Ohlson VM: Folk medicine in the health practice of Hmong refugees.  West J Nurs Res 1988, 10: 647-660. H2.
Cherniack EP, Ceron-Fuentes J, Florez H, Sandals L, Rodriguez O, Palacios JC: Influence of race and ethnicity on alternative medicine as a self-treatment preference for common medical conditions in a population of multi-ethnic urban elderly. Complement Ther Clin Pract 2008, 14: 116-123. H2.
Cheung CK, Wyman JF, Halcon LL: Use of complementary and alternative therapies in community-dwelling older adults. J Altern Complement Med 2007, 13: 997-1006. H2.
Cho BH: The politics of herbal drugs in Korea. Soc Sci Med 2000, 51: 505-509. N3.
Chomat AMB, Wilson IB, Wanke CA, Selvakumar A, John KR, Isaac R: Knowledge, Beliefs, and Health Care Practices Relating to Treatment of HIV in Vellore, India. Aids Patient Care and Stds 2009, 23: 477-484. L3.
Chomitz KM: At loggerheads? Agricultural expansion, poverty reduction and environment in the tropical forests. Washington: World Bank Policy Research Report; 2007. N1, N2.
Chotchoungchatchai S, Saralamp P, Jenjittikul T, Pornsiripongse S, Prathanturarug S: Medicinal plants used with Thai Traditional Medicine in modern healthcare services: a case study in Kabchoeng Hospital, Surin Province, Thailand. J Ethnopharmacol 2012, 141: 193-205. L2.
Choudhry UK: Traditional practices of women from India: pregnancy, childbirth, and newborn care. J Obstet Gynecol Neonatal Nurs 1997, 26: 533-539. L3.
Cizmesija T, Bergman-Markovic B: [Use of complementary and alternative medicine among the patients in primary health care]. Acta Med Croatica 2008, 62: 15-22. H2.
Clarke DB, Doel MA, Segrott J: No alternative? The regulation and professionalization of complementary and alternative medicine in the United Kingdom. Health Place 2004, 10: 329-338. L2.
Cocks M, Dold A: The role of 'African chemists' in the health care system of the Eastern Cape province of South Africa. Soc Sci Med 2000, 51: 1505-1515. H2.
Cocks M, Moller V: Use of indigenous and indigenised medicines to enhance personal well-being: a South African case study. Soc Sci Med 2002, 54: 387-397. H2.
Cohen MH: Regulation, religious experience, and epilepsy: a lens on complementary therapies. Epilepsy Behav 2003, 4: 602-606. L2.
Cohen RJ, Ek K, Pan CX: Complementary and alternative medicine (CAM) use by older adults: a comparison of self-report and physician chart documentation. J Gerontol A Biol Sci Med Sci 2002, 57: M223-M227. H2.
Connett GJ, Lee BW: Treating childhood asthma in Singapore: when West meets East. BMJ 1994, 308: 1282-1284. L3.
Cook C, Baisden D: Ancillary use of folk medicine by patients in primary care clinics in southwestern West Virginia. South Med J 1986, 79: 1098-1101. H2.
Cordell GA: Sustainable medicines and global health care. Planta Medica 2011, 77:1129-1138. H2, H3, L2.
Cosminsky S: Women and health care on a Guatemalan plantation. Soc Sci Med 1987, 25: 1163-1173. H2.
Coulibaly A, Gbary R, Le BJ, Rey JL: [Utilization of modern and traditional health systems in the rural Ivory Coast area]. Ann Soc Belg Med Trop 1989, 69: 331-336. H2.
Cox PA: Will tribal knowledge survive the millennium? Science 2000, 287: 44-45. L1.
Cramer H, Shaw A, Wye L, Weiss M: Over-the-counter advice seeking about complementary and alternative medicines (CAM) in community pharmacies and health shops: an ethnographic study. Health & Social Care in the Community 2010, 18: 41-50. H2.
Crandon L: Grass roots, herbs, promotors and preventions: a re-evaluation of contemporary international health care planning. The Bolivian case. Soc Sci Med 1983, 17: 1281-1289. N3.
Crawford NW, Cincotta DR, Lim A, Powell CV: A cross-sectional survey of complementary and alternative medicine use by children and adolescents attending the University Hospital of Wales. BMC Complement Altern Med 2006, 6: 16. H2.
Crawford SY, Manuel AM, Wood BD: Pharmacists' considerations when serving Amish patients. J Am Pharm Assoc 2009, 49: 86-94. L2.
Critchley LA, Chen DQ, Lee A, Thomas GN, Tomlinson B: A survey of Chinese herbal medicine intake amongst preoperative patients in Hong Kong. Anaesth Intensive Care 2005, 33: 506-513. L3.
Cuellar NG, Rogers AE, Hisghman V: Evidenced based research of complementary and alternative medicine (CAM) for sleep in the community dwelling older adult. Geriatr Nurs 2007, 28: 46-52. H2, L3.
Cuzzolin L, Zaffani S, Murgia V, Gangemi M, Meneghelli G, Chiamenti G et al.: Patterns and perceptions of complementary/alternative medicine among paediatricians and patients' mothers: a review of the literature. Eur J Pediatr 2003, 162: 820-827. L2, H2.
Cuzzolin L, Zaffani S, Benoni G: Safety implications regarding use of phytomedicines. Eur J Clin Pharmacol 2006, 62: 37-42. H2.
D'souza M: Health and indigenous development. Health Millions 1993, 1: 6-7. L2.
Damgaard-Morch NL, Nielsen LJ, Uldall SW: [Knowledge and perceptions of complementary and alternative medicine among medical students in Copenhagen]. Ugeskr Laeger 2008, 170: 3941-3945. L2.
Dangor T, Ross E: Western and traditional medicine: cultural beliefs and practices of South African Muslims with regard to Down syndrome. S Afr J Commun Disord 2006, 53: 27-38. H2.
Daramola SO: The Nigerian patient: his choice for traditional treatment. Niger Nurse 1978, 10: 22-23. H2.
Das FA, Barua I, Das DD: Ethno-Medicinal Practices: A Case Study among the Sonowal Kacharis of Dibrugarh, Assam.  Studies on Ethno-Medicine 2008, 2: 33-37. H2.
Davidson J: The survival of traditional medicine in a Peruvian barriada. Soc Sci Med 1983, 17: 1271-1280. H2, H3, H4.
Davidson P, Hancock K, Leung D, Ang E, Chang E, Thompson DR et al.: Traditional Chinese Medicine and heart disease: what does Western medicine and nursing science know about it? Eur J Cardiovasc Nurs 2003, 2: 171-181. L2.
Davies-Adetugbo AA: Sociocultural factors and the promotion of exclusive breastfeeding in rural Yoruba communities of Osun State, Nigeria. Soc Sci Med 1997, 45: 113-125. L3.
Davis EW, Yost JA: The ethnomedicine of the Waorani of Amazonian Ecuador. J Ethnopharmacol 1983, 9: 273-297. H2.
de Medeiros PM, Soldati GT, Alencar NL, Vandebroek I, Pieroni A, Hanazaki N et al.: The use of medicinal plants by migrant people: adaptation, maintenance, and replacement. Evid Based Complement Alternat Med 2012, 2012: 807452. H2, H4.
de Melo JG, de Amorim EL, de Albuquerque UP: Native medicinal plants commercialized in Brazil - priorities for conservation. Environ Monit Assess 2009, 156: 567-580. L1.
de Ridder S, van der Kooy F, Verpoorte R: Artemisia annua as a self-reliant treatment for malaria in developing countries. Journal of Ethnopharmacology 2008, 120: 302-314. I2, L3.
de LL-J, Rendon AB: Traditional knowledge among Zapotecs of Sierra Madre Del Sur, Oaxaca. Does it represent a base for plant resources management and conservation? J Ethnobiol Ethnomed 2012, 8: 24. L1.
de Z, I, Carson D, Feachem R, Kirkwood B, Lindsay-Smith E, Loewenson R: Perceptions of childhood diarrhoea and its treatment in rural Zimbabwe. Soc Sci Med 1984, 19: 727-734. H2, L3.
del Mundo WF, Shepherd WC, Marose TD: Use of alternative medicine by patients in a rural family practice clinic.  Fam Med 2002, 34: 206-212. H2.
Delgado M: Herbal medicine in the Puerto Rican community. Health Soc Work 1979, 4: 24-40. L2.
Delgoda R, Ellington C, Barrett S, Gordon N, Clarke N, Younger N: The practice of polypharmacy involving herbal and prescription medicines in the treatment of diabetes mellitus, hypertension and gastrointestinal disorders in Jamaica. West Indian Med J 2004, 53: 400-405. L3.
Delgoda R, Younger N, Barrett C, Braithwaite J, Davis D: The prevalence of herbs use in conjunction with conventional medicines in Jamaica. Complement Ther Med 2010, 18: 13-20. H2.
Desmarchelier C, Gurni A, Ciccia G, Giukietti AM: Ritual and medicinal plants of the Ese'jas of the Amazonian rainforest (Madre de Dios, Perú). Journal of Ethnopharmacology 1996, 52:45-51. L2.
Develay A, Sauerborn R, Diesfeld HJ: Utilization of health care in an African urban area: results from a household survey in Ouagadougou, Burkina Faso. Social Science and Medicine 1996, 43:1611-1619. H2, H4.
Dhalla S, Chan KJ, Montaner JS, Hogg RS: Complementary and alternative medicine use in British Columbia--a survey of HIV positive people on antiretroviral therapy. Complement Ther Clin Pract 2006, 12: 242-248. L3.
Diallo D, Diakite C, Mounkoro PP, Sangare D, Graz B, Falquet J et al.: [Knowledge of traditional healers on malaria in Kendi (Bandiagara) and Finkolo (Sikasso) in Mali ]. Mali Med 2007, 22: 1-8. L2.
Diallo D, Diakite C, Diawara A, Konate N, Teme S, Giani S: [Study of the consumption of the Improved Traditional Phytomedicines in the health district of Kadiolo (Region of Sikasso, Mali)]. Mali Med 2010, 25: 5-13. H2.
Diallo D, Graz B, Falquet J, Traoré AK, Giani S, Mounkoro PP, Berthé A, Sacko M, Diakité C: Malaria treatment in remote areas of Mali: use of modern and traditional medicines, patient outcome. Transactions of the Royal Society of Tropical Medicine and Hygiene 2006, 100:515-520. L2.
Digby A: Self-medication and the trade in medicine within a multi-ethnic context: a case study of South Africa from the mid-nineteenth to mid-twentieth centuries. Social History of Medicine 2005, 18:439-457. H2.
Dillard JN, Knapp S: Complementary and alternative pain therapy in the emergency department. Emerg Med Clin North Am 2005, 23: 529-549. H2.
Disayavanish C, Disayavanish P: Introduction of the treatment method of Thai traditional medicine: its validity and future perspectives. Psychiatry Clin Neurosci 1998, 52 Suppl: S334-S337. N3.
Dlamini CS, Geldenhuys CJ: The socioeconomic status of the non-timber forest product subsector in Swaziland. Southern Forests 2009, 71: 311-318. H3.
Doel MA, Segrott J: Materializing complementary and alternative medicine: aromatherapy, chiropractic, and Chinese herbal medicine in the UK. Geoforum 2004, 35: 727-738. H2.
Dourma M, Wala K, Bellefontaine R, Batawila K, Guelly KA, Akpagana K: A Comparative Analysis of Resource Use and Regeneration in Two Types of Open Isoberlinia Woodland in Togo. Bois et Forets des Tropiques 2009, 5-19. L1.
Druss BG, Rosenheck RA: Association between use of unconventional therapies and conventional medical services. Journal of the American Medical Association 1999, 282:651-656. H2.
Duleba K, Wysocki M, Styczynski J: [Physicians attitudes towards complementary and alternative medicine in patients with cancer: preliminary report from pediatric and oncology centers]. Med Wieku Rozwoj 2008, 12: 1148-1154. L2.
Dzator J, Asafu-Adjaye J: A study of malaria care provider choice in Ghana. Health Policy 2004, 69: 389-401. L3.
Eberhardie C: Nutritional supplements and the EU: is anyone happy? Proceedings of the Nutrition Society 2007, 66: 508-511. N3.
Ebi KL, Woodruff R, Hildebrand AV, Corvalan C: Climate change-related health impacts in the Hindu Kush-Himalayas. EcoHealth 2007, 4:264-270. N4.
Eddouks M, Maghrani M, Lemhadri A, Ouahidi ML, Jouad H: Ethnopharmacological survey of medicinal plants used for the treatment of diabetes mellitus, hypertension and cardiac diseases in the south-east region of Morocco (Tafilalet). J Ethnopharmacol  2002, 82: 97-103. L3.
Edirne T, Arica SG, Gucuk S, Yildizhan R, Kolusari A, Adali E et al.: Use of complementary and alternative medicines by a sample of Turkish women for infertility enhancement: a descriptive study. BMC Complement Altern Med 2010, 10: 11. L3.
Edman JL, Koon TY: Mental illness beliefs in Malaysia: ethnic and intergenerational comparisons. Int J Soc Psychiatry 2000, 46: 101-109. L3.
Eigner D, Scholz D: Ferula asa-foetida and Curcuma longa in traditional medical treatment and diet in Nepal. J Ethnopharmacol 1999, 67: 1-6. H2.
Eilu G, Oriekot J, Tushabe H: Conservation of indigenous plants outside protected areas in Tororo District, eastern Uganda. African Journal of Ecology 2007, 45: 73-78. L1.
Einterz EM: Perceptions of malaria transmission, presentation and management in northern Cameroon. Trans R Soc Trop Med Hyg 2003, 97: 51-52. L3.
Eisenberg DM, Davis RB, Ettner SL, Appel S, Wilkey S, Rompay MV, Kessler RC: Trends in alternative medicine use in the United States, 1990-1997: results of a follow-up national survey. Journal of the American Medical Association 1998, 280:1569-1575. H2.
EMA: Action plan for herbal medicines 2010-2011. London: European Medicines Agency, EMA/831327/2009; 2010. N3.
Eng J, Ramsum D, Verhoef M, Guns E, Davison J, Gallagher R: A population-based survey of complementary and alternative medicine use in men recently diagnosed with prostate cancer. Integr Cancer Ther 2003, 2: 212-216. H2, L3.
Ref ID: 743
Eriksson K: [Obstetric help in the village of Karve in Nepal]. Jordemodern 1994, 107: 122-123. L2.
Erlichman J, Salam A, Haber BA: Use of complementary and alternative medicine in pediatric chronic viral hepatitis. J Pediatr Gastroenterol Nutr 2010, 50: 417-421. L2.
Ernst E: Prevalence of use of complementary/alternative medicine: a systematic review. Bulletin of the World Health Organization 2000, 78:252-257. H2, L3.
Ernst E, Fugh-Berman A: Complementary and alternative medicine: what is it all about? Occupational and Environmental Medicine 2002, 59:140-144. H2, L3.
Evans A, Duncan B, McHugh P, Shaw J, Wilson C: Inpatients' use, understanding, and attitudes towards traditional, complementary and alternative therapies at a provincial New Zealand hospital. N Z Med J 2008, 121: 21-34. H2.
Fabian E, Toscher S, Elmadfa I, Pieber TR:  Use of Complementary and Alternative Medicine Supplements in Patients with Diabetes Mellitus. Annals of Nutrition and Metabolism 2011, 58: 101-108. L3.
Fahimi F, Hrgovic I, El-Safadi S, Munstedt K: Complementary and alternative medicine in obstetrics: a survey from Iran. Arch Gynecol Obstet 2011, 284: 361-364. L2.
Farnes C, Beckstrand RL, Callister LC: Help-seeking behaviours in childbearing women in Ghana, West Africa. Int Nurs Rev 2011, 58: 491-497. H2.
Federici E, Multari G, Gallo FR, Palazzino G: [Herbal drugs: from traditional use to regulation]. Ann Ist Super Sanita 2005, 41: 49-54. N3.
Feldmann JM, Wiemann CM, Sever L, Hergenroeder AC: Folk and traditional medicine use by a subset of Hispanic adolescents. Int J Adolesc Med Health 2008, 20: 41-51. H2.
Feng L, Chiam PC, Kua EH, Ng TP: Use of complementary and alternative medicines and mental disorders in community-living Asian older adults. Arch Gerontol Geriatr 2010, 50: 243-249. L3.
Ferro MA, Leis A, Doll R, Chiu L, Chung M, Barroetavena MC: The impact of acculturation on the use of traditional Chinese medicine in newly diagnosed Chinese cancer patients. Support Care Cancer 2007, 15: 985-992. L3.
Fifanou VG, Ousmane C, Gauthier B, Brice S: Traditional agroforestry systems and biodiversity conservation in Benin (West Africa). Agroforestry Systems 2011, 82: 1-13. L1.
Fikree FF, Ali T, Durocher JM, Rahbar MH: Health service utilization for perceived postpartum morbidity among poor women living in Karachi. Soc Sci Med 2004, 59: 681-694. H2, L3.
Filshie J, Rubens CN: Complementary and alternative medicine. Anesthesiol Clin 2006, 24: 81-111. L2.
Finerman RD: Experience and expectation: conflict and change in traditional family health care among the Quichua of Saraguro. Soc Sci Med 1983, 17: 1291-1298. L2.
Flatie T, Gedif T, Asres K, Gebre-Mariam T: Ethnomedical survey of Berta ethnic group Assosa Zone, Benishangul-Gumuz regional state, mid-west Ethiopia. J Ethnobiol Ethnomed 2009, 5: 14. H2.
Flores FP, Umenai T, Wakai S: Should community-managed drug stores be phased out? Asia Pac J Public Health 2001, 13: 9-12. L2.
Foller ML: A new approach to community health. Soc Sci Med 1989, 28: 811-818. L2.
Foster GM: How to get well in Tzintzuntzan. Soc Sci Med 1985, 21: 807-818. H2.
Frate DA, Croom EM, Jr., Frate JB, Juergens JP, Meydrech EF: Use of plant-derived therapies in a rural, biracial population in Mississippi. J Miss State Med Assoc 1996, 37: 427-429. H2.
Freymann H, Rennie T, Bates I, Nebel S, Heinrich M: Knowledge and use of complementary and alternative medicine among British undergraduate pharmacy students. Pharm World Sci 2006, 28: 13-18. L2.
Fries CJ: Classification of complementary and alternative medical practices: Family physicians' ratings of effectiveness. Can Fam Physician 2008, 54: 1570-1571. L2.
Froment A: Biodiversity, environment and health among rainforest-dwellers: an evolutionary perspective. In Human health and forests. Edited by Colfer CJP. London: Earthscan; 2008:259-273. L2.
Fu YN, Brookfield H, Guo HJ, Chen J, Chen AG, Cui JY: Smallholder rubber plantation expansion and its impact on local livelihoods, land use and agrobiodiversity, a case study from Daka, Xishuangbanna, southwestern China. International Journal of Sustainable Development and World Ecology 2009, 16: 22-29. N2.
Fukuda S, Watanabe E, Ono N, Tsubouchi M, Shirakawa T: [Use of complementary and alternative medicine and health problems]. Nihon Koshu Eisei Zasshi 2006, 53: 293-300. H2.
Gardner JM, Grant D, Hutchinson S, Wilks R: The use of herbal teas and remedies in Jamaica. West Indian Med J 2000, 49: 331-335. H2.
Garrow D, Egede LE: National patterns and correlates of complementary and alternative medicine use in adults with diabetes. J Altern Complement Med 2006, 12: 895-902. H2, L3.
Gbolade AA: Inventory of antidiabetic plants in selected districts of Lagos State, Nigeria. J Ethnopharmacol 2009, 121: 135-139. L2.
Gedif T, Hahn HJ: Treatment of malaria in Ethiopian folk medicine. Trop Doct 2002, 32: 206-209. L2.
Gedif T, Hahn HJ: The use of medicinal plants in self-care in rural central Ethiopia. Journal of Ethnopharmacology 2003, 87: 155-161. H2.
Geissler PW, Nokes K, Prince RJ, Odhiambo RA, Aagaard-Hansen J, Ouma JH: Children and medicines: self-treatment of common illnesses among Luo schoolchildren in western Kenya. Soc Sci Med 2000, 50: 1771-1783. H2.
Geist HJ, Lambin EF: Proximate causes and underlying driving forces of tropical deforestation. BioScience 2002, 52:143-150. N1.
Gesler WM: Therapeutic landscapes: medical issues in light of the new cultural geography. Soc Sci Med 1992, 34: 735-746. H2. 
Ghassemi J: Finding the evidence in CAM: a student's perspective. Evidence-Based Complementary and Alternative Medicine 2005, 2: 395-397. L2.
Ghimire SK, McKey D, Aumeeruddy-Thomas Y: Conservation of Himalayan medicinal plants: Harvesting patterns and ecology of two threatened species, Nardostachys grandiflora DC. and Neopicrorhiza scrophulariiflora (Pennell) Hong. Biological Conservation 2005, 124:463-475. H5, H6.
Giannelli M, Cuttini M, Arniani S, Baldi P, Buiatti E: [Non-conventional medicine in Tuscany: attitudes and use in the population]. Epidemiol Prev 2004, 28: 27-33. H2.
Giovannini P, Reyes-Garcia V, Waldstein A, Heinrich M: Do pharmaceuticals displace local knowledge and use of medicinal plants? Estimates from a cross-sectional study in a rural indigenous community, Mexico. Soc Sci Med 2011, 72: 928-936. L2. 
Glover DD, Amonkar M, Rybeck BF, Tracy TS:  Prescription, over-the-counter, and herbal medicine use in a rural, obstetric population. Am J Obstet Gynecol 2003, 188: 1039-1045. H2.
Gohar F, Greenfield SM, Beevers DG, Lip GY, Jolly K: Self-care and adherence to medication: a survey in the hypertension outpatient clinic. BMC Complement Altern Med 2008, 8: 4. L3.
Gold EB, Bair Y, Zhang G, Utts J, Greendale GA, Upchurch D et al.: Cross-sectional analysis of specific complementary and alternative medicine (CAM) use by racial/ethnic group and menopausal status: the Study of Women's Health Across the Nation (SWAN). Menopause 2007, 14: 612-623. H2, L3.
Gollschewski S, Anderson D, Skerman H, Lyons-Wall P: The use of complementary and alternative medications by menopausal women in South East Queensland. Womens Health Issues 2004, 14: 165-171. L3.
Gomez-Martinez R, Tlacuilo-Parra A, Garibaldi-Covarrubias R: Use of complementary and alternative medicine in children with cancer in Occidental, Mexico. Pediatr Blood Cancer 2007, 49: 820-823. L3.
Gorn SB, Navarro SA, Solano NS: The use of alternative and complementary therapies on the Mexican population with depressive and anxiety disorders: results of a survey in Mexico City. Salud Mental 2009, 32: 107-115. H2, L3.
Gorter AC, Sanchez G, Pauw J, Perez RM, Sandiford P, Smith GD: [Childhood diarrhea in rural Nicaragua: beliefs and traditional health practices]. Bol Oficina Sanit Panam 1995, 119: 377-390. H2.
Goswami M, Dash B, Dash NC: Traditional Method of Reproductive Health Care Practices and Fertility Control among the Bhumija Tribe of Baleswar, Orissa. Studies on Ethno-Medicine 2011, 5: 51-55. H2.
Grabbe L: Understanding patients from the former Soviet Union. Fam Med 2000, 32: 201-206. H2.
Graham RE, Ahn AC, Davis RB, O'Connor BB, Eisenberg DM, Phillips RS: Use of complementary and alternative medical therapies among racial and ethnic minority adults: results from the 2002 National Health Interview Survey. J Natl Med Assoc 2005, 97: 535-545. H2.
Green EC, Makhubu L: Traditional healers in Swaziland: toward improved cooperation between the traditional and modern health sectors. Soc Sci Med 1984, 18: 1071-1079. L2.
Greenfield S, Pattison H, Jolly K: Use of complementary and alternative medicine and self-tests by coronary heart disease patients. Bmc Complementary and Alternative Medicine 2008, 8. L3.
Grzywacz JG, Arcury TA, Bell RA, Lang W, Suerken CK, Smith SL et al.: Ethnic differences in elders' home remedy use: sociostructural explanations. Am J Health Behav 2006, 30: 39-50. H2.
Guarrera PM, Lucia LM: Ethnobotanical remarks on Central and Southern Italy. J Ethnobiol Ethnomed 2007, 3: 23. H2.
Gums JG, Carson DS: Influence of folk medicine on the family practitioner. South Med J 1987, 80: 209-212. L2.
Habermann TM, Thompson CA, LaPlant BR, Bauer BA, Janney CA, Clark MM et al.: Complementary and alternative medicine use among long-term lymphoma survivors: a pilot study. Am J Hematol 2009, 84: 795-798. L3.
Halberstein RA, Saunders AB: Traditional medical practices and medicinal plant usage on a Bahamian island. Cult Med Psychiatry 1978, 2: 177-203. H2.
Hales S, Kovats S, Woodward A: What El Niño can tell us about human health and global climate change. Global Change and Human Health 2000, 1:66-77. I2.
Hall HG, Griffiths DL, McKenna LG: The use of complementary and alternative medicine by pregnant women: a literature review. Midwifery 2011, 27: 817-824. L3.
Hall HG, McKenna LG, Griffiths DL: Midwives' support for Complementary and Alternative Medicine: a literature review. Women Birth 2012, 25: 4-12. L2.
Hamilton JL, Roemheld-Hamm B, Young DM, Jalba M, DiCicco-Bloom B: Complementary and alternative medicine in US family medicine practices: a pilot qualitative study. Altern Ther Health Med 2008, 14: 22-27. H2.
Hamilton WR, Monaghan MS, Turner PD: Comparison of pharmacy practitioner and pharmacy student attitudes toward complementary and alternative therapies in a rural state. American Journal of Pharmaceutical Education 2002, 66: 55-58. L2.
Han GS: The provision of hanbang herbal medicine in the Korean community in Australia: entrepreneurial or caring for fellow Koreans? Aust Health Rev 2001, 24: 146-155. L2.
Han GS, Ballis H: Ethnomedicine and dominant medicine in multicultural Australia: a critical realist reflection on the case of Korean-Australian immigrants in Sydney. J Ethnobiol Ethnomed 2007, 3: 1. L2, H2, H4.
Handelman L, Rich M, Bridgemohan CF, Schneider L: Understanding pediatric inner-city asthma: an explanatory model approach. J Asthma 2004, 41: 167-177. L3.
Harrigan JT: Patient disclosure of the use of complementary and alternative medicine to their obstetrician/gynaecologist. J Obstet Gynaecol 2011, 31: 59-61. H2.
Harris IM, Kingston RL, Rodriguez R, Choudary V: Attitudes towards complementary and alternative medicine among pharmacy faculty and students. Am J Pharm Educ 2006, 70: 129. L2.
Harris P, Rees R: The prevalence of complementary and alternative medicine use among the general population: a systematic review of the literature. Complementary Therapies in Medicine 2000, 8:88-96. H2.
Hartini TN, Padmawati RS, Lindholm L, Surjono A, Winkvist A: The importance of eating rice: changing food habits among pregnant Indonesian women during the economic crisis. Soc Sci Med 2005, 61: 199-210. H2.
Hasan SS, Ahmed SI, Bukhari NI, Loon WC: Use of complementary and alternative medicine among patients with chronic diseases at outpatient clinics. Complement Ther Clin Pract 2009, 15: 152-157. H2, L3.
Hastings-Tolsma M, Terada M: Complementary medicine use by nurse midwives in the U.S. Complement Ther Clin Pract 2009, 15: 212-219. L2.

Hay SI, Guerra CA, Tatem AJ, Atkinson PM, Snow RW: Urbanization, malaria transmission and disease burden in Africa. Nature Reviews 2005, 3:81-90. L3.

Heckler SL: Herbalism, home gardens, and hybridization: Wothiha medicine and cultural change. Med Anthropol Q 2007, 21: 41-63. H2.

Hecht S, Kandel S, Gomes I, Cuellar N, Rosa H: Globalization, forest resurgence, and environmental politics in El Salvador. World Development 2006, 34:308-323. I5, N6.
Heinrich M: Herbal and Symbolic Medicines of the Lowland Mixe (Oaxaca, Mexico) - Disease Concepts, Healers Roles, and Plant Use. Anthropos 1994, 89: 73-83. L2, H2.
Hersch-Martínez P: Medicinal plants and regional traders in Mexico: Physiographic differences and conservational challenge. Economic Botany 1997, 51:107-120. L1.
Herman CJ, Allen P, Hunt WC, Prasad A, Brady TJ: Use of complementary therapies among primary care clinic patients with arthritis. Prev Chronic Dis 2004, 1: A12. L3.
Hess S, De GS, Halter K, Dickenmann M, Denhaerynck K: Prevalence and correlates of selected alternative and complementary medicine in adult renal transplant patients. Clin Transplant 2009, 23: 56-62. L3.
Heuschkel R, Afzal N, Wuerth A, Zurakowski D, Leichtner A, Kemper K et al.: Complementary medicine use in children and young adults with inflammatory bowel disease. Am J Gastroenterol 2002, 97: 382-388. L3.
Hielscher S, Sommerfeld J: Concepts of illness and the utilization of health-care services in a rural Malian village. Soc Sci Med 1985, 21: 469-481. H2.
Hilgert NI, Gil GE: Changes in the use of the environment and herbal medicines. Case study in the Argentine Yungas. Boletin Latinoamericano y del Caribe de Plantas Medicinales y Aromaticas 2008, 7: 130-140. H4.
Hilgert NI, Higuera MDL, Kristensen MJ: Herbal medicine in the urban context. Case study in a neighborhood of the city of Tandil, Argentina. Boletin Latinoamericano y del Caribe de Plantas Medicinales y Aromaticas 2010, 9: 177-190. H2, H4.
Hilsden RJ, Meddings JB, Verhoef MJ: Complementary and alternative medicine use by patients with inflammatory bowel disease: An Internet survey. Can J Gastroenterol 1999, 13: 327-332. L3.
Hilsden RJ, Verhoef MJ, Best A, Pocobelli G: Complementary and alternative medicine use by Canadian patients with inflammatory bowel disease: results from a national survey. Am J Gastroenterol 2003, 98: 1563-1568. H2.
Himmel W, Schulte M, Kochen MM: Complementary medicine: are patients' expectations being met by their general practitioners? Br J Gen Pract 1993, 43: 232-235. L2, H2.
Ho NK: Understanding traditional Chinese medicine--a doctor's viewpoint. Singapore Med J 2001, 42: 487-492. L2, H2.
Holliday I: Traditional medicines in modern societies: an exploration of integrationist options through East Asian experience. J Med Philos 2003, 28: 373-389. N3.
Homma AKO: Modernisation and technological dualism in the extractive economy in Amazonia. In Current Issues in Non-Timber Forest Products Research. Edited by Ruiz-Pérez M, Arnold JEM. Bogor: Center for International Forestry Research; 1996:59-82. L1.
Homsy J: The availability of local and affordable treatments for AIDS in Sub-Saharan Africa. J Altern Complement Med 1999, 5: 505-507. L2.
Homsy J, King R, Tenywa J, Kyeyune P, Opio A, Balaba D: Defining minimum standards of practice for incorporating African traditional medicine into HIV/AIDS prevention, care, and support: a regional initiative in Eastern and Southern Africa. Journal of Alternative and Complementary Medicine 2004, 10:905-910. L2.
Hon KL, Twinn SF, Leung TF, Thompson DR, Wong Y, Fok TF: Chinese nursing students' attitudes toward traditional Chinese medicine. J Nurs Educ 2006, 45: 182-185. L2.
Hongoro C, Musonza TG, Macq J, Anozie A: A qualitative assessment of the referral system at district level in Zimbabwe: implications on efficiency and effective delivery of health services. Cent Afr J Med 1998, 44: 93-97.L2, H2.
Hossen A, Westhues A: In search of healing between two worlds: the use of traditional and modern health services by older women in rural Bangladesh. Soc Work Health Care 2012, 51: 327-344. H2.
Hsu MC, Creedy D, Moyle W, Venturato L, Tsay SL, Ouyang WC: Use of Complementary and Alternative Medicine among adult patients for depression in Taiwan. J Affect Disord  2008, 111: 360-365. L3.
Hsu MC, Moyle W, Creedy D, Venturato L, Ouyang WC, Tsay SL: Use of antidepressants and complementary and alternative medicine among outpatients with depression in Taiwan. Arch Psychiatr Nurs 2009, 23: 75-85. L3.
Hu T: The financing and the economic efficiency of rural health services in the People's Republic of China. Int J Health Serv 1976, 6: 239-249. N3.
Huber FK, Ineichen R, Yang Y, Weckerle CS: Livelihood and conservation aspects of non-wood forest product collection in the Shaxi Valley, southwest China. Economic Botany 2010, 64:189-204. L1.
Huber R, Koch D, Beiser I, Zschocke I, Luedtke R: Experience and attitudes towards CAM--a survey of internal and psychosomatic patients in a German university hospital. Altern Ther Health Med 2004, 10: 32-36. H2, L3.
Hughes GD, Puoane TR, Clark BL, Wondwossen TL, Johnson Q, Folk W: Prevalence and Predictors of Traditional Medicine Utilization Among Persons Living with Aids (Plwa) on Antiretroviral (Arv) and Prophylaxis Treatment in Both Rural and Urban Areas in South Africa. African Journal of Traditional Complementary and Alternative Medicines 2012, 9: 470-484. H2, L3.
Hunt K, Ernst E: Evidence-based practice in British complementary and alternative medicine: double standards? J Health Serv Res Policy 2009, 14:  219-223. N3.
Idowu OA, Mafiana CF, Luwoye IJ, Adehanloye O: Perceptions and home management practices of malaria in some rural communities in Abeokuta, Nigeria. Travel Med Infect Dis 2008, 6: 210-214. L3.
Idowu OA, Mafiana CF, Sotiloye D: Traditional birth home attendance and its implications for malaria control during pregnancy in Nigeria. Trans R Soc Trop Med Hyg 2008, 102: 679-684. H2, L3.
Ikoh MU, Udo AU, Charles AO, Charles JO: The influence of "stock out" on health-seeking behavior of low income women in Uyo urban, Akwa Ibom State, Nigeria. Int Q Community Health Educ 2008, 29: 257-273. H2, L2.
Imperato PJ, Traore D: Traditional beliefs about measles and its treatment among the Bambara of Mali. Trop Geogr Med 1969, 21: 62-67. L3.
Imperato PJ: The role of women in traditional healing among the Bambara of Mali. Trans R Soc Trop Med Hyg 1981, 75: 766-770. H2.
Israelsen LD: The challenge of regulation, globalization and climate change on botanicals and traditional medicines: Respecting tradition while embracing change. Planta Medica 2008, 74: 318-319. I1, N3.
Iwu MM: African medicinal plants in the search for new drugs based on ethnobotanical leads. Ciba Found Symp 1994, 185: 116-126. N3.
Izugbara CO, Afangideh AI: Urban women's use of rural-based health care services: the case of Igbo women in Aba City, Nigeria. Journal of Urban Health 2005, 82:111-121. L2.
Jacobsson L, Merdasa F: Traditional perceptions and treatment of mental disorders in western Ethiopia before the 1974 revolution. Acta Psychiatr Scand 1991, 84: 475-481. L3.
Janes CR: The health transition, global modernity and the crisis of traditional medicine: the Tibetan case. Soc Sci Med 1999, 48: 1803-1820. N3, L3.
Jarman CN, Perron BE, Kilbourne AM, Teh CF: Perceived treatment effectiveness, medication compliance, and complementary and alternative medicine use among veterans with bipolar disorder. J Altern Complement Med 2010, 16: 251-255. L3.
Jezewski MA, Poss J: Mexican Americans' explanatory model of type 2 diabetes. West J Nur Res 2002, 24: 840-858. L3.
Johannessen H, von Bornemann HJ, Pasquarelli E, Fiorentini G, Di CF, Miccinesi G: Prevalence in the use of complementary medicine among cancer patients in Tuscany, Italy. Tumori 2008, 94: 406-410. L3, L4.
Johnson SK, Blanchard A: Alternative medicine and herbal use among university students. Journal of American College Health 2006, 55: 163-168. H2.
Jones RA, Taylor AG, Bourguignon C, Steeves R, Fraser G, Lippert M et al.: Complementary and alternative medicine modality use and beliefs among African American prostate cancer survivors. Oncol Nurs Forum 2007, 34: 359-364. L3.
Joos S, Musselmann B, Szecsenyi J: Integration of complementary and alternative medicine into family practices in Germany: results of a national survey. Evid Based Complement Alternat Med 2011, 2011: 495813. L2.
Jorgensen TM, Andersson KA, Mardby AC: Beliefs about medicines among Swedish pharmacy employees. Pharm World Sci 2006, 28: 233-238. L2.
Junaid R, Abaas M, Fatima B, Anis I, Hussain M: Attitude and Practice of Patients and Doctors towards Complementary and Alternative Medicine. Journal of the Pakistan Medical Association 2012, 62: 865-868. L2.
Ka V: Hard choices: the use of Western vs. Chinese traditional medicine by the Chinese homebound elderly, New York City. A community health survey. J Long Term Home Health Care 1998, 17: 2-10. H2.
Kalasagar M, Sivapathasundharam B, Einstein TB: AIDS awareness in an Indian metropolitan slum dweller: a KAP (knowledge, attitude, practice) study. Indian J Dent Res 2006, 17: 66-69. L3.
Kamalanathan JP: Comparative study on the acceptance and use of contraceptive methods in a rural population in Kelantan. Malays J Reprod Health 1990, 8: 66-71. L3.
Kanodia AK, Legedza AT, Davis RB, Eisenberg DM, Phillips RS: Perceived benefit of Complementary and Alternative Medicine (CAM) for back pain: a national survey. J Am Board Fam Med 2010, 23: 354-362. L3.
Karadeniz C, Pinarli FG, Oguz A, Gursel T, Canter B: Complementary/alternative medicine use in a pediatric oncology unit in Turkey. Pediatr Blood Cancer 2007, 48: 540-543. L3.
Karimi K: The impact of current alternative herbal remedies on dental patient management. Gen Dent 1999, 47: 264-266. L2.
Kauchali S, Rollins N, Van den Broeck J: Local beliefs about childhood diarrhoea: importance for healthcare and research. J Trop Pediatr 2004, 50: 82-89. L3.
Kay AB, Lessof MH: Allergy: conventional and alternative concepts. A report of the Royal College of Physicians Committee on Clinical Immunology and Allergy. Clinical and Experimental Allergy 1992, 22(Suppl):i-44. H2.
Kayombo EJ, Uiso FC, Mahunnah RLA: Experience on healthcare utilization in seven administrative regions of Tanzania. Journal of Ethnobiology and Ethnomedicine 2012, 8:5. L2, H2.
Keegan L: Use of alternative therapies among Mexican Americans in the Texas Rio Grande Valley. J Holist Nurs 1996, 14: 277-294. H2, H4.
Kemper KJ, Vincent EC, Scardapane JN: Teaching an integrated approach to complementary, alternative, and mainstream therapies for children: a curriculum evaluation. J Altern Complement Med 1999, 5: 261-268. L2.
Kerdpon D, Sriplung H: Factors related to delay in diagnosis of oral squamous cell carcinoma in southern Thailand. Oral Oncol 2001, 37: 127-131. H2, L3.
Kerr RB, Berti PR, Chirwa M: Breastfeeding and mixed feeding practices in Malawi: timing, reasons, decision makers, and child health consequences. Food Nutr Bull 2007, 28: 90-99. L3.
Keshet Y, Ben-Arye E: [Which complementary and alternative medicine modalities are integrated within Israeli healthcare organizations and do they match the public's preferences?]. Harefuah 2011, 150: 635-8, 690, 689. H2, L2.
Kessler RC, Davis RB, Foster DF, Rompay MIV, Walters EE, Wilkey SA, Kaptchuk TJ, Eisenberg DM: Long-term trends in the use of complementary and alternative medical therapies in the United States. Annals of Internal Medicine 2001, 135:262-268. H2.
Kim JS, Yoon SS: Perspectives of stroke in persons living in Seoul, South Korea. A survey of 1000 subjects. Stroke 1997, 28: 1165-1169. L3.
Kim S, Hohrmann JL, Clark S, Munoz KN, Braun JE, Doshi A et al.: A multicenter study of complementary and alternative medicine usage among ED patients. Acad Emerg Med 2005, 12: 377-380. H2, L3.
King R: Collaboration with traditional healers in HIV/AIDS prevention and care in sub-Saharan Africa. Geneva: UNAIDS; 2000. L2.
Klein JD, Wilson KM, Sesselberg TS, Gray NJ, Yussman S, West J: Adolescents' knowledge of and beliefs about herbs and dietary supplements: a qualitative study. J Adolesc Health 2005, 37: 409. H2.
Klunklin A, Greenwood J: "Hanging in" with HIV/AIDS in the rural north of Thailand: a grounded theory study. J Assoc Nurses AIDS Care 2005, 16: 24-32. L3.
Knoss W, Stolte F, Reh K: [The regulatory framework for complementary and alternative medicines in Europe]. Bundesgesundheitsblatt Gesundheitsforschung Gesundheitsschutz 2008, 51: 771-778. N3.
Knotek K, Verner V, Chaloupkova P, Kokoska L: Prevalence and use of herbal products in the Czech Republic: over-the-counter survey among adult pharmacies clients. Complement Ther Med 2012, 20: 199-206. H2.
Koc Z, Topatan S, Saglam Z: Use of and attitudes toward complementary and alternative medicine among midwives in Turkey. Eur J Obstet Gynecol Reprod Biol 2012, 160: 131-136. L2.
Kofi-Tsekpo M: Institutionalization of African traditional medicine in health care systems in Africa. Afr J Health Sci 2004, 11: i-ii. N3.
Koh HL, Teo HH, Ng HL: Pharmacists' patterns of use, knowledge, and attitudes toward complementary and alternative medicine. J Altern Complement Med 2003, 9: 51-63. L2.
Kroesen K, Baldwin CM, Brooks AJ, Bell IR:  US military veterans' perceptions of the conventional medical care system and their use of complementary and alternative medicine. Family Practice 2002, 19: 57-64. H2.
Kronenberg F, Mindes J, Jacobson JS: The future of complementary and alternative medicine for cancer. Cancer Invest 2005, 23: 420-426. L3.
Krosch SL: Perceptions and use of complementary and alternative medicine in American Samoa: a survey of health care providers. Hawaii Med J 2010, 69: 21-26. L2.
Kuan YC, Yen DJ, Yiu CH, Lin YY, Kwan SY, Chen C et al.: Treatment-seeking behavior of people with epilepsy in Taiwan: A preliminary study. Epilepsy & Behavior 2011, 22: 308-312. H2, L3.
Kulig JC: Conception and birth control use: Cambodian refugee women's beliefs and practices. J Community Health Nurs 1988, 5: 235-246. L3.
Kulig JC: Childbearing beliefs among Cambodian refugee women. West J Nurs Res 1990, 12: 108-118. L3.
Kumar GP, Kumar R, Chaurasia OP, Singh SB:  Current status and potential prospects of medicinal plant sector in trans-Himalayan Ladakh. Journal of Medicinal Plants Research 2011, 5: 2929-2940. L1.
Kumar M, Sheikh MA, Bussmann RW: Ethnomedicinal and ecological status of plants in Garhwal Himalaya, India. J Ethnobiol Ethnomed 2011, 7: 32. L1.
Kusimba J, Voeten HA, O'Hara HB, Otido JM, Habbema JD, Ndinya-Achola JO et al.: Traditional healers and the management of sexually transmitted diseases in Nairobi, Kenya. Int J STD AIDS 2003, 14: 197-201. L2, L4.
LaCaille RA, Kuvaas NJ: Coping styles and self-regulation predict complementary and alternative medicine and herbal supplement use among college students. Psychol Health Med 2011, 16: 323-332. H2.
Ladinsky JL, Volk ND, Robinson M: The influence of traditional medicine in shaping medical care practices in Vietnam today. Soc Sci Med 1987, 25: 1105-1110. N3.
Lai D, Chappell N: Use of Traditional Chinese Medicine by older Chinese immigrants in Canada. Fam Pract 2007, 24: 56-64. H2, H4.
Lai DW, Surood S: Chinese health beliefs of older Chinese in Canada. J Aging Health 2009, 21: 38-62. H2.
Lakatos L, Czegledi Z, David G, Kispal Z, Kiss LS, Palatka K et al.: [Treatment adherence and use of complementary and alternative medicine in patients with inflammatory bowel disease]. Orv Hetil 2010, 151: 250-258. H2, L3.
Lakatos PL, Czegledi Z, David G, Kispal Z, Kiss LS, Palatka K et al.: Association of adherence to therapy and complementary and alternative medicine use with demographic factors and disease phenotype in patients with inflammatory bowel disease. J Crohns Colitis 2010, 4: 283-290. H2.
Lambert J, Srivastava J, Vietmayer N: Medicinal plants – rescuing a global heritage. Technical Paper no. 355. Washington DC: World Bank; 1997. L1.
Lambin EF, Turner II BL, Geist HJ, Agbola SB, Angelsen A, Bruce JW, Coomes O, Dirzo R, Fischer G, Folke C, George PS, Homewood K, Imbernon J, Leemans R, Li X, Moran EF, Mortimore M, Ramakrishnan PS, Richards MB, Skånes H, Steffen WL, Stone GD, Svedin U, Veldkamp TA, Vogel C, Xu J: The causes of land-use and land-cover change: moving beyond the myths. Global Environmental Change 2001, 11:261-269. N2.
Lamorde M, Tabuti JR, Obua C, Kukunda-Byobona C, Lanyero H, Byakika-Kibwika P et al.: Medicinal plants used by traditional medicine practitioners for the treatment of HIV/AIDS and related conditions in Uganda. J Ethnopharmacol 2010, 130: 43-53. L2.
Langlois-Klassen D, Kipp W, Rubaale T: Who's talking? Communication between health providers and HIV-infected adults related to herbal medicine for AIDS treatment in western Uganda. Social Science & Medicine 2008, 67: 165-176. L2.
Langwick S: From Non-Aligned Medicines to Market-Based Herbals: China's Relationship to the Shifting Politics of Traditional Medicine in Tanzania. Medical Anthropology 2010, 29: 15-43. N3.
Larsen HO, Olsen CS: Unsustainable collection and unfair trade? Uncovering and assessing assumptions regarding Central Himalayan medicinal plant conservation. Biodiversity and Conservation 2007, 16: 1679-1697. L1.
Lazarus M: Marketing herbal medicines. AIDS Action 1999, 6. L2.
Le GA, Sri-Ngernyuang L, Streefland PH: Enhancing appropriate drug use: the contribution of herbal medicine promotion. A case study in rural Thailand. Soc Sci Med 1993, 36: 1023-1035. L2, H2.
Leach MA, Fairhead JR, Millimouno D, Diallo AA: New therapeutic landscapes in Africa: parental categories and practices in seeking infant health in the Republic of Guinea. Soc Sci Med 2008, 66: 2157-2167. L3.
Lee MS, Lim HJ, Lee MS, Jang HS: Perceptions, knowledge and misuse of an oriental herbal drug: a survey of 608 Korean female nursing college students. Complement Ther Clin Pract 2005, 11: 200-204. L2.
Lee MR: Is it really medicine? The traditional and alternative medicine act and informal health economy in the Philippines. Asia Pac J Public Health 2009, 21: 333-345. N3.
Lee RP: Health services system in Hong Kong: professional stratification in a modernizing society. Inquiry 1975, 12: 51-62. L2.
Lee WC: Medical education and medical practice in Korea. J Med Educ 1970, 45: 283-292. L2.
Lee YK, Georgiou C, Raab C: The knowledge, attitudes, and practices of dietitians licensed in Oregon regarding functional foods, nutrient supplements, and herbs as complementary medicine. J Am Diet Assoc 2000, 100: 543-548. L2.
Lemay JF, Amin A, Pacaud D: Complementary and alternative medicine use in children and adolescents with type 1 diabetes. Paediatrics & Child Health 2011, 16: 468-472. L3.
Lenaerts M: Substances, relationships and the omnipresence of the body: an overview of Asheninka ethnomedicine (Western Amazonia). J Ethnobiol Ethnomed 2006, 2: 49. H2.
Leonard KL: African traditional healers and outcome-contingent contracts in health care. Journal of Development Economics 2003, 71:1-22. L2.
Leong EM, Semple SJ, Angley M, Siebert W, Petkov J, McKinnon RA: Complementary and alternative medicines and dietary interventions in multiple sclerosis: what is being used in South Australia and why? Complement Ther Med 2009, 17: 216-223. L3.
Leslie C: What caused India's massive community health workers scheme: a sociology of knowledge. Soc Sci Med 1985, 21: 923-930. N3.
Lewis WH: Pharmaceutical discoveries based on ethnomedicinal plants: 1985 to 2000 and beyond. Economic Botany 2003, 57:126-134. I4.
Li CP: A new medical trend in China. Am J Chin Med (Gard City N Y) 1975, 3: 213-221. L2.
Li L: [My recognition on integrated traditional Chinese and Western medicine]. Zhongguo Zhong Xi Yi Jie He Za Zhi 1998, 18: 707-708. L1.
Li LY, Ciren BZ, Zhan D, Wei YF: [Comprehensive utilization and development of traditional Tibetan medicine in China]. Zhongguo Zhong Yao Za Zhi 2001, 26: 808-810. L1.
Li XQ, Yang JN, Su YH: [Does the utilization of traditional Chinese herbs threaten the biodiversity?]. Zhong Xi Yi Jie He Xue Bao 2007, 5: 363-367. L1.
Liang X, Feng J, Jin Y, Guo Z, Xu Q: [Prospects of the development of quality control technologies for traditional Chinese medicine]. Se Pu 2008, 26: 130-135. L2.
Lim MK, Sadarangani P, Chan HL, Heng JY: Complementary and alternative medicine use in multiracial Singapore. Complementary Therapies in Medicine 2005, 13:16-24. H2, L3.
Lim J, Wong M, Chan MY, Tan AM, Rajalingam V, Lim LP et al.: Use of complementary and alternative medicine in paediatric oncology patients in Singapore. Ann Acad Med Singapore 2006, 35: 753-758. L3.
Lin YH, Chen KK, Chiu JH: Prevalence, patterns, and costs of Chinese medicine use among prostate cancer patients: a population-based study in Taiwan. Integr Cancer Ther 2010, 9: 16-23. L3.
Lin YH, Chiu JH: Use of Chinese medicine by women with breast cancer: a nationwide cross-sectional study in Taiwan. Complement Ther Med 2011, 19: 137-143. H2, L3.
Lin YH, Chen KK, Chiu JH: Trends in Chinese medicine use among prostate cancer patients under national health insurance in Taiwan: 1996-2008. Integr Cancer Ther 2011, 10: 317-327. L2, L3.
Liu C, Yang Y, Gange SJ, Weber K, Sharp GB, Wilson TE et al.: Disclosure of complementary and alternative medicine use to health care providers among HIV-infected women. AIDS Patient Care STDS 2009, 23: 965-971. H2, L3.
Lock M: Licorice in leviathan: the medicalization of care for the Japanese elderly. Cult Med Psychiatry 1984, 8: 121-139. L2.
Loera JA, Black SA, Markides KS, Espino DV, Goodwin JS: The use of herbal medicine by older Mexican Americans. Journals of Gerontology Series A-Biological Sciences and Medical Sciences 2001, 56: M714-M718. H2.
Loera JA, Reyes-Ortiz C, Kuo YF: Predictors of complementary and alternative medicine use among older Mexican Americans. Complement Ther Clin Pract 2007, 13: 224-231. H2.
Loh CH: Use of traditional Chinese medicine in Singapore children: perceptions of parents and paediatricians. Singapore Med J 2009, 50: 1162-1168. L2, L3.
Loman DG: The use of complementary and alternative health care practices among children. J Pediatr Health Care 2003, 17: 58-63. H2.
Losier A, Taylor B, Fernandez CV: Use of alternative therapies by patients presenting to a pediatric Emergency Department. Journal of Emergency Medicine 2005, 28: 267-271. H2, L3.
Lun KC, Ho CS, Ng CH: The role of Chinese traditional medical practice as a form of health care in Singapore--IV. Physicians in private practice. Am J Chin Med 1983, 11: 43-53. L2.
Luque JS: Healthcare choices and acute respiratory infection: A rural Ecuadorian case study. Human Organization 2007, 66: 282-291. L2, L3.
Ma K, Lee SS, Chu EK, Tam DK, Kwong VS, Ho CF et al.: Popular use of traditional Chinese medicine in HIV patients in the HAART era. AIDS Behav 2008, 12: 637-642. L3.
Mackenzie ER, Taylor L, Bloom BS, Hufford DJ, Johnson JC: Ethnic minority use of complementary and alternative medicine (CAM): a national probability survey of CAM utilizers. Altern Ther Health Med 2003, 9: 50-56. H2.
Madsen H, Andersen S, Nielsen RG, Dolmer BS, Host A, Damkier A: Use of complementary/alternative medicine among paediatric patients. Eur J Pediatr 2003, 162: 334-341. L3.
Mafimisebi TE, Oguntade AE: Preparation and use of plant medicines for farmers' health in Southwest Nigeria: socio-cultural, magico-religious and economic aspects. J Ethnobiol Ethnomed 2010, 6: 1. H2.
Mahabir D, Gulliford MC: Use of medicinal plants for diabetes in Trinidad and Tobago. Rev Panam Salud Publica 1997, 1: 174-179. L3.
Mahat TBS, Griffin DM, Shepherd KR: Human impact on some forests of the middle hills of Nepal. 1. Forestry in the context of the traditional resources of the state. Mountain Research and Development 1986, 6:223-232. N6.
Malak AT, Karayurt O, Demir E, Yumer AS: Complementary and alternative medicine in cancer patients - analysis of influencing factors in Turkey. Asian Pac J Cancer Prev 2009, 10: 1083-1087. L3.
Manahan L, Caragay R, Muirden KD, Allander E, Valkenburg HA, Wigley RD: Rheumatic pain in a Philippine village. A WHO-ILAR COPCORD Study. Rheumatol Int 1985, 5: 149-153. L3.
Manaseki S: Mongolia: a health system in transition. BMJ 1993, 307: 1609-1611. L2, H2.
Manjkhola S, Dhar U: Conservation and utilization of Arnebia benthamii (Wall. ex G. Don) Johnston - a high value Himalayan medicinal plant. Current Science 2002, 83: 484-488. L1.
Mansky PJ, Wallerstedt DB: Complementary medicine in palliative care and cancer symptom management. Cancer J 2006, 12: 425-431. L2.
Manya K, Champion B, Dunning T: The use of complementary and alternative medicine among people living with diabetes in Sydney. BMC Complement Altern Med 2012, 12: 2. L3.
Marbella AM, Harris MC, Diehr S, Ignace G, Ignace G: Use of Native American healers among Native American patients in an urban Native American health center. Arch Fam Med 1998, 7: 182-185. H2.
Marian F, Widmer M, Herren S, Donges A, Busato A: Physicians' philosophy of care: a comparison of complementary and conventional medicine. Forsch Komplementmed 2006, 13: 70-77. L2.
Marian F: Complementary medicine: equity issues in evaluation and policy-making. Forsch Komplementmed 2007, 14 Suppl 2: 2-9. N3.
Marriott BM, Campbell L, Hirsch E, Wilson D: Preliminary data from demographic and health surveys on infant feeding in 20 developing countries. J Nutr 2007, 137: 518S-523S. L3.
Martin HA, Hull TH, Preston-Whyte E, Bagnol B, Smit J, Wacharasin C et al.: A cross cultural study of vaginal practices and sexuality: implications for sexual health. Soc Sci Med 2010, 70: 392-400. L3.
Martin KJ, Jordan TR, Vassar AD, White DB: Herbal and nonherbal alternative medicine use in Northwest Ohio. Ann Pharmacother 2002, 36: 1862-1869. H2.
Martinez H, Saucedo G: Mothers' perceptions about childhood diarrhoea in rural Mexico. J Diarrhoeal Dis Res 1991, 9: 235-243. L3.
Martinez H, Suriano K, Ryan GW, Pelto GH:  [Ethnography of acute respiratory infections in a rural zone of Mexican highlands]. Salud Publica Mex 1997, 39: 207-216. L3.
Maskarinec G, Shumay DM, Kakai H, Gotay CC: Ethnic differences in complementary and alternative medicine use among cancer patients. J Altern Complement Med 2000, 6: 531-538. H2, L3.
Matthe DS: Ethnomedical science and African medical practice. Med Law 1989, 7: 517-521. H2.
Mbura JS, Mgaya HN, Heggenhougen HK: The use of oral herbal medicine by women attending antenatal clinics in urban and rural Tanga District in Tanzania. East Afr Med J 1985, 62: 540-550. L3.
McCabe P: Natural therapies in Australia: a nurse-naturopath's view. Nurse Pract Forum 1994, 5: 114-117. L1.
McCormick LH: A few things I've learned about CAM. Med Econ 2003, 80: 89-90. L2.
Mccrea CE, Pritchard ME: Concurrent herb-prescription medication use and health care provider disclosure among university students. Complementary Therapies in Medicine 2011, 19: 32-36. H2.
McIntyre M: Can CAM come in from the cold? An update on the ongoing regulatory process with regard to herbal and acupuncture practitioners in the United Kingdom. J Altern Complement Med 2003, 9: 809-810. N3.
McKenzie JL, Chrisman NJ: Healing herbs, gods, and magic: folk health beliefs among Filipino-Americans. Nurs Outlook 1977, 25: 326-329. H2.
McMichael AJ, Woodruff RE, Hales S: Climate change and human health: present and future risks. Lancet 2006, 367:859-869. I2.
Mcmillen H, Scheinman D: Using herbs. AIDS Action 1999, 5. L2.
McNaughton C, Eidsness LM: Ethics of alternative therapies. S D J Med 1995, 48: 209-211. L2, H2.
McPherson F, Schwenka MA: Use of complementary and alternative therapies among active duty soldiers, military retirees, and family members at a military hospital. Mil Med 2004, 169: 354-357. H2.
Mega TP, Santos PD, Souza-Machado A, Noblat LDBC, Cruz AA: Use of medicinal herbs by patients with severe asthma managed at a Referral Center. Brazilian Journal of Pharmaceutical Sciences 2011, 47: 643-649. H2, L3.
Mehta DH, Phillips RS, Davis RB, McCarthy EP: Use of complementary and alternative therapies by Asian Americans. Results from the National Health Interview Survey. J Gen Intern Med 2007, 22: 762-767. H2, H4.
Melchart D, Linde K, Weidenhammer W, Hager S, Liao JZ, Bauer R et al.: Use of traditional drugs in a hospital of Chinese medicine in Germany. Pharmacoepidemiology and Drug Safety 1999, 8: 115-120. L2.
Mendoza RL: Is It Really Medicine? The Traditional and Alternative Medicine Act and Informal Health Economy in the Philippines. Asia-Pacific Journal of Public Health 2009, 21: 333-345. N3.
Meng XJ, Liu SH, Wang P: [Comprehensive utilization and sustainable development of Chinese herbal medicinal resource]. Zhongguo Zhong Xi Yi Jie He Za Zhi 2008, 28: 463-465. L1.
Meng XZ, Su YH, Zhu DZ: [Sustainable utilization of Radix Glycyrrhizae for protection of ecology environment and herbal resources]. Zhong Xi Yi Jie He Xue Bao 2006, 4: 556-559. L1.
Micozzi MS: Anthropological study of health beliefs, behaviors, and outcomes: traditional folk medicine and ethnopharmacology. Hum Organ 1983, 42: 351-353. H2.
Mihalynuk TV, Knopp RH, Scott CS, Coombs JB: Physician informational needs in providing nutritional guidance to patients. Family Medicine 2004, 36: 722-726. L2.
Mikhail BI: Hispanic mothers' beliefs and practices regarding selected children's health problems. West J Nurs Res 1994, 16: 623-638. L3.
Miles A: Science, nature, and tradition: the mass-marketing of natural medicine in urban Ecuador.  Med Anthropol Q 1998, 12: 206-225. H2.
Miller JL, Binns HJ, Brickman WJ: Complementary and alternative medicine use in children with type 1 diabetes: a pilot survey of parents. Explore (NY) 2008, 4: 311-314. H2, L3.
Miller NN, Strickler JC: China's revolution in health. Am Univ Field Staff Rep Asia 1980, 3: 1-24. N3.
Miller NN: Traditional medicine in East Africa: the search for a synthesis. Am Univ Field Staff Rep Afr 1980, 22: 1-15. N3.
Mills SY: The House of Lords report on complementary medicine: a summary. Complement Ther Med 2001, 9: 34-39. N3.
Misra R, Balagopal P, Klatt M, Geraghty M: Complementary and alternative medicine use among Asian Indians in the United States: a national study. J Altern Complement Med 2010, 16: 843-852. H2, H4.
Mitchell MF: Popular medical concepts in Jamaica and their impact on drug use. West J Med 1983, 139: 841-847. L3.
Molares S, Ladio A: Mapuche perceptions and conservation of Andean Nothofagus forests and their medicinal plants: a case study from a rural community in Patagonia, Argentina. Biodiversity and Conservation 2012, 21: 1079-1093. L1.
Molassiotis A, Margulies A, Fernandez-Ortega P, Pud D, Panteli V, Bruyns I et al.: Complementary and alternative medicine use in patients with haematological malignancies in Europe. Complement Ther Clin Pract 2005, 11: 105-110. L3.
Molassiotis A, Fernadez-Ortega P, Pud D, Ozden G, Scott JA, Panteli V et al.: Use of complementary and alternative medicine in cancer patients: a European survey. Ann Oncol 2005, 16: 655-663. L3.
Molassiotis A, Ozden G, Platin N, Scott JA, Pud D, Fernandez-Ortega P et al.: Complementary and alternative medicine use in patients with head and neck cancers in Europe. Eur J Cancer Care (Engl) 2006, 15: 19-24. L3.
Molassiotis A, Panteli V, Patiraki E, Ozden G, Platin N, Madsen E et al.: Complementary and alternative medicine use in lung cancer patients in eight European countries. Complement Ther Clin Pract 2006, 12: 34-39. L3.
Molina I, Luxardo N: Nonconventional therapies in cancer. Medicina 2005, 65:390-394. H2.
Monteiro JM, de Almeida CF, de Albuquerque UP, de Lucena RF, Florentino AT, de Oliveira RL: Use and traditional management of Anadenanthera colubrina (Vell.) Brenan in the semi-arid region of northeastern Brazil. J Ethnobiol Ethnomed 2006, 2: 6. L1.
Morgan RW: Migration as a factor in the acceptance of medical care. Soc Sci Med 1973, 7: 865-873. H3.
Morris B: Herbalism and divination in southern Malawi. Soc Sci Med 1986, 23: 367-377. H2.
Moss K, Boon H, Ballantyne P, Kachan N: The professionalization of Western herbalists: response to new product regulations in Canada. Complement Ther Med 2007, 15: 264-270. N3.
Msuya TS, Kideghesho JR: The role of traditional management practices in enhancing sustainable use and conservation of medicinal plants in West Usambara Mountains, Tanzania. Tropical Conservation Science 2009, 2: 88-105. L1.
Mukherjee PK: Exploring botanicals in Indian system of medicine - Regulatory perspectives. Clinical Research and Regulatory Affairs 2003, 20: 249-264. N1.
Mulaudzi FM, Makhubela-Nkondo ON: Indigenous healers' beliefs and practices concerning sexually transmitted diseases. Curationis 2006, 29: 46-53. L2.
Munguti KJ: Community perceptions and treatment seeking for malaria in Baringo district, Kenya: implications for disease control. East Afr Med J 1998, 75: 687-691. H2, L3.
Nagai SC, Queiroz MS: [Alternative and complementary medicine in the basic health system network in Brazil: a qualitative approach]. Cien Saude Colet 2011, 16: 1793-1800. L2.
Nagata JM, Jew AR, Kimeu JM, Salmen CR, Bukusi EA, Cohen CR: Medical pluralism on Mfangano Island: use of medicinal plants among persons living with HIV/AIDS in Suba District, Kenya. Journal of Ethnopharmacology 2011, 135:501-509. H2.
Nanayakkara V, Ekanayake L: Use of traditional medicine for oral conditions in rural Sri Lanka. Int Dent J 2008, 58: 86-90. H2, L3.
Nations MK, Misago C, Fonseca W, Correia LL, Campbell OM: Women's hidden transcripts about abortion in Brazil. Soc Sci Med 1997, 44: 1833-1845. H2, L3.
Ndulo J, Faxelid E, Krantz I: Traditional healers in Zambia and their care for patients with urethral/vaginal discharge. J Altern Complement Med 2001, 7: 529-536. L2.
Nearing M: The green pharmacy. Herbal medicines in modern usage. IDRC Rep 1985, 14: 10-11. L1.
Nebelkopf E: Herbal therapy in the treatment of drug use. Int J Addict 1987, 22: 695-717. H2, L3.
Neiberg RH, Aickin M, Grzywacz JG, Lang W, Quandt SA, Bell RA et al.: Occurrence and co-occurrence of types of complementary and alternative medicine use by age, gender, ethnicity, and education among adults in the United States: the 2002 National Health Interview Survey (NHIS). J Altern Complement Med 2011, 17: 363-370. H2.
Nelson GC, Bennett E, Berhe AA, Cassman K, DeFries R, Dietz T, Dobermann A, Dobson A, Janetos A, Levy M, Marco D, Nakicenovic N, O'Neill B, Norgaard R, Petschel-Held G, Ojima D, Pingali P, Watson R, Zurek M: Anthropogenic drivers of ecosystem change: an overview. Ecology and Society 2006, 11:29. N6.
Ness J, Cirillo DJ, Weir DR, Nisly NL, Wallace RB: Use of complementary medicine in older Americans: results from the Health and Retirement Study. Gerontologist 2005, 45: 516-524. H2.
Neuman RP, Hirsch E: Commercialisation of non-timber forest products: review and analysis of research. Bogor: Center for International Forestry Research; 2000. H3.
Newton JR, Santangeli L, Shakeel M, Ram B: Use of complementary and alternative medicine by patients attending a rhinology outpatient clinic. Am J Rhinol Allergy 2009, 23: 59-63. H2, L3.
Ngang PN, Ntaganira J, Kalk A, Wolter S, Ecks S: Perceptions and beliefs about cough and tuberculosis and implications for TB control in rural Rwanda. Int J Tuberc Lung Dis 2007, 11: 1108-1113. H2, L3.
Ni H, Simile C, Hardy AM: Utilization of complementary and alternative medicine by United States adults: results from the 1999 national health interview survey. Med Care 2002, 40: 353-358. H2.
Nicholson T: Complementary and alternative medicines (including traditional Maori treatments) used by presenters to an emergency department in New Zealand: a survey of prevalence and toxicity. N Z Med J 2006, 119: U1954. H2.
Nissen N: Practitioners of Western herbal medicine and their practice in the UK: beginning to sketch the profession. Complement Ther Clin Pract 2010, 16: 181-186. L2.
Njoroge GN, Kibunga JW: Herbal medicine acceptance, sources and utilization for diarrhoea management in a cosmopolitan urban area (Thika, Kenya). African Journal of Ecology 2007, 45: 65-70. H2, L3.
Njoroge GN, Kaibui IM, Njenga PK, Odhiambo PO: Utilisation of priority traditional medicinal plants and local people's knowledge on their conservation status in arid lands of Kenya (Mwingi District). J Ethnobiol Ethnomed 2010, 6: 22. L1.
Nowack R, Balle C, Birnkammer F, Koch W, Sessler R, Birck R: Complementary and alternative medications consumed by renal patients in southern Germany. J Ren Nutr 2009, 19: 211-219. H2, L3.
Nuttall P, Flores FC: Hmong healing practices used for common childhood illnesses. Pediatr Nurs 1997, 23: 247-251. H2, L3.
Nwankwo BO, Brieger WR: Exclusive breastfeeding is undermined by use of other liquids in rural southwestern Nigeria. J Trop Pediatr 2002, 48: 109-112. H2.
Nwoga IA: Traditional healers and perceptions of the causes and treatment of cancer. Cancer Nurs 1994, 17: 470-478. L2.
Nwoke BE: Behavioural aspects and their possible uses in the control of dracontiasis (guinea-worm) in Igwun river basin area of Imo State, Nigeria. Angew Parasitol 1992, 33: 205-210. L3, H2.
Nyika A: Ethical and regulatory issues surrounding African traditional medicine in the context of HIV/AIDS. Dev World Bioeth 2007, 7: 25-34. N3.
O'Dempsey TJ: Traditional belief and practice among the Pokot people of Kenya with particular reference to mother and child health: 1. The Pokot people and their environment. Ann Trop Paediatr 1988, 8: 49-60. L4.
Obisesan KA, Adeyemo AA: Infertility and other fertility related issues in the practice of traditional healers and Christian religious healers in south western Nigeria. Afr J Med Med Sci 1998, 27: 51-55. L3.
Ohlendorf W: Domestication and crop development of Duboisia spp. (Solanacea). In Domestication and commercialization of non-timber forest products in agroforestry systems. Edited by Leakey RRB, Temu AB, Melnyk M, Vantomme P. Rome: FAO; 1996:183-187. L1.
Oke GA, Bankole OO, Denloye OO, Danfillo IS, Enwonwu CO: Traditional and emerging oral health practices in parts of Nigeria. Odontostomatol Trop 2011, 34: 35-46. L3.
Okyere E, Tawiah-Agyemang C, Manu A, Deganus S, Kirkwood B, Hill Z: Newborn care: the effect of a traditional illness, asram, in Ghana. Ann Trop Paediatr 2010, 30: 321-328. L3.
Olago D, Marshall M, Wandiga SO, Opondo M, Yanda PZ, Kanalawe R, Githeko AK, Downs T, Opere A, Kavumvuli R, Kirumira E, Ogallo L, Mugambi P, Apindi E, Githui F, Kathuri J, Olaka L, Sigalla R, Nanyunja R, Baguma T, Achola P: Climatic, socio-economic, and health factors affecting human vulnerability to cholera in the Lake Victoria basin, East Africa. Ambio 2007, 36:350-358. H3, L2, N4.
Olsen CS, Helles F: Market efficiency and benefit distribution in medicinal plant markets: empirical evidence from South Asia. International Journal of Biodiversity Science and Management 2009, 5:53-62. H3.
Olsen SA: A review of complementary and alternative medicine (CAM) by people with multiple sclerosis. Occup Ther Int 2009, 16: 57-70. L3.
Ong CO, Chan LY, Yung PB, Leung TN: Use of traditional Chinese herbal medicine during pregnancy: a prospective survey. Acta Obstet Gynecol Scand 2005, 84: 699-700. H2, L3.
Opala J, Boillot F: Leprosy among the Limba: illness and healing in the context of world view. Soc Sci Med 1996, 42: 3-19. L2.
Orhan F, Sekerel BE, Kocabas CN, Sackesen C, Adalioglu G, Tuncer A: Complementary and alternative medicine in children with asthma. Ann Allergy Asthma Immunol 2003, 90: 611-615. H2, L3.
Osaka R, Nanakorn S: Health care of villagers in northeast Thailand--a health diary study. Kurume Med J 1996, 43: 49-54. H2, L3.
Osamor PE, Owumi BE: Complementary and alternative medicine in the management of hypertension in an urban Nigerian community. BMC Complement Altern Med 2010, 10: 36. H2, H3.
Oshikoya KA, Senbanjo IO, Njokanma OF, Soipe A: Use of complementary and alternative medicines for children with chronic health conditions in Lagos, Nigeria. Bmc Complementary and Alternative Medicine 2008, 8. L3.
Ostrom E: Governing the commons: The evolution of institutions for collective action (political economy of institutions and decisions). New York: Cambridge University Press; 1990. L1.
Osungbade KO, Siyanbade SL: Myths, misconceptions, and misunderstandings about epilepsy in a Nigerian rural community: implications for community health interventions. Epilepsy Behav 2011, 21: 425-429. L3.
Osuntokun BO: The traditional basis of neuropsychiatric practice among the Yorubas of Nigeria. Trop Geogr Med 1975, 27: 422-430. L2.
Ottolini MC, Hamburger EK, Loprieato JO, Coleman RH, Sachs HC, Madden R et al.: Complementary and alternative medicine use among children in the Washington, DC area. Ambul Pediatr 2001, 1: 122-125. H2, L3.
Park Y: [Japan's Oriental medicine policy in colonial Korea]. Uisahak 2008, 17: 75-86. N3.
Parry ML, Canziani OF, Palutikof JP, van der Linden PJ, Hanson CE: Contribution of working group II to the fourth assessment report of the Intergovernmental Panel on Climate Change. Cambridge: Cambridge University Press; 2007. I1, I3.
Parry ML, Rosenzweig C, Iglesias A, Livermore M, Fischer G: Effects of climate change on global food production under SRES emissions and socio-economic scenarios. Global Environmental Change 2004, 4:53-67. I3.
Peebles CT, McAuley JW, Roach J, Moore JL, Reeves AL: Alternative Medicine Use by Patients with Epilepsy. Epilepsy Behav 2000, 1: 74-77. L2.
Pei SJ: Ethnobotanical approaches of traditional medicine studies: Some experiences from Asia. Pharmaceutical Biology 2001, 39: 74-79. L1.
Peltzer K, Preez NF, Ramlagan S, Fomundam H: Use of traditional complementary and alternative medicine for HIV patients in KwaZulu-Natal, South Africa. BMC Public Health 2008, 8: 255. H2, L3.
Pena JC: [The concept of illness and kidney diseases in Nahuatl medicine. Synthesis of Mesoamerican pre-Columbian medicine]. Rev Invest Clin 2002, 54: 474-481. L3.
Peroni N, Martins PS: Influence of shift cultivation dynamics on the creation of diversity of ethnovarieties propagated as clones. Interciencia 2000, 25: 22-29. L1.
Perry B, Gesler W: Physical access to primary health care in Andean Bolivia. Social Science and Medicine 2000, 50:1177-1188. L2.
Perurena FC: [Relationship between gender and holistic representations of health and illness]. Rev Gaucha Enferm 1997, 18: 104-112. H2.
Pesek T, Abramiuk M, Garagic D, Fini N, Meerman J, Cal V: Sustaining plants and people: traditional Q'eqchi' Maya botanical knowledge and interactive spatial modeling in prioritizing conservation of medicinal plants for culturally relative holistic health promotion. Ecohealth 2009, 6: 79-90. L1.
Petit C, Scudder T, Lambin E: Quantifying processes of land-cover change by remote sensing: resettlement and rapid land-cover changes in south–eastern Zambia. International Journal of Remote Sensing 2001, 22:3435-3456. N1.
Picking D, Younger N, Mitchell S, Delgoda R: The prevalence of herbal medicine home use and concomitant use with pharmaceutical medicines in Jamaica. J Ethnopharmacol 2011, 137: 305-311. H2, L3.
Pieroni A, Quave CL: Traditional pharmacopoeias and medicines among Albanians and Italians in southern Italy: A comparison. Journal of Ethnopharmacology 2005, 101: 258-270. H2.
Pieroni A, Torry B: Does the taste matter? Taste and medicinal perceptions associated with five selected herbal drugs among three ethnic groups in West Yorkshire, Northern England. J Ethnobiol Ethnomed 2007, 3: 21. H2.
Pitetti R, Singh S, Hornyak D, Garcia SE, Herr S: Complementary and alternative medicine use in children. Pediatr Emerg Care 2001, 17: 165-169. L3.
Planta M, Gundersen B, Petitt JC: Prevalence of the use of herbal products in a low-income population. Fam Med 2000, 32: 252-257. H2.
Poss JE, Jezewski MA, Stuart AG: Home remedies for type 2 diabetes used by Mexican Americans in El Paso, Texas.  Clin Nurs Res 2003, 12: 304-323. L3.
Pouliot M: Relying on nature's pharmacy in rural Burkina Faso: Empirical evidence of the determinants of traditional medicine consumption. Social Science and Medicine 2011, 73:1498-1507. H2, L3.
Pud D, Kaner E, Morag A, Ben-Ami S, Yaffe A: Use of complementary and alternative medicine among cancer patients in Israel. Eur J Oncol Nurs 2005, 9: 124-130. H2, L3.
Quah TC: In Response To: Professor Cassileth's manuscript on "Alternative and Complementary Cancer Treatments," Featured in The Oncologist 1996;1:173-179. Oncologist 1996, 1: 324-325. L2, L3.
Quan H, Lai D, Johnson D, Verhoef M, Musto R: Complementary and alternative medicine use among Chinese and white Canadians. Can Fam Physician 2008, 54: 1563-1569. H2, L3.
Quimby EL: The use of herbal therapies in pediatric oncology patients: treating symptoms of cancer and side effects of standard therapies. J Pediatr Oncol Nurs 2007, 24: 35-40. L3.
Quinlan MB, Quinlan RJ: Modernization and medicinal plant knowledge in a Caribbean horticultural village. Med Anthropol Q 2007, 21: 169-192. H2, H3.
Ramos-Remus C, Gamez-Nava JI, Gonzalez-Lopez L, Skeith KJ, Perla-Navarro AV, Galvan-Villegas F et al.: Use of alternative therapies by patients with rheumatic disease in Guadalajara, Mexico: Prevalence, beliefs, and expectations. Arthritis Care and Research 1998, 11: 411-418. H2, L3.
Ramos-Remus C, Raut A: Complementary and alternative practices in rheumatology. Best Practice & Research in Clinical Rheumatology 2008, 22: 741-757. L3.
Rana MS, Samant SS: Population of Lilium polyphyllum D. Dom ex Royle – A critically endangered medicinal plant in a protected area of Northwestern Himalaya. Journal for Nature Conservation 2011, 19:137-142. H5, H6.
Ransford HE, Carrillo FR, Rivera Y: Health care-seeking among Latino immigrants: blocked access, use of traditional medicine, and the role of religion. J Health Care Poor Underserved 2010, 21: 862-878. H2, H4, L2.
Rao MR, Palada MC, Becker BN: Medicinal and aromatic plants in agroforestry systems. Agroforestry Systems 2004, 61-2: 107-122. L1.
Rauyajin O, Pasandhanatorn V, Rauyajin V, Na-nakorn S, Ngarmyithayapong J, Varothai C: Mothers' hygiene behaviours and their determinants in Suphanburi, Thailand. J Diarrhoeal Dis Res 1994, 12: 25-34. L3.
Raynal C, Lefebvre T: [Ayurvedics drugs in France. Laboratories polytherapic, a test]. Rev Hist Pharm (Paris) 2011, 58: 413-430. L2.
Redzic SS: The ecological aspect of ethnobotany and ethnopharmacology of population in Bosnia and Herzegovina. Coll Antropol 2007, 31: 869-890. H2, L3.
Reeve M: Concepts of illness and treatment practice in a caboclo community of the lower Amazon. Medical Anthropology Quarterly 2000, 14: 96-108. L3.
Reiff M, O'Connor B, Kronenberg F, Balick M, Lohr P, Roble M et al.: Ethnomedicine in the urban environment: Dominican healers in New York City. Human Organization 2003, 62: 12-26. L2.
Reznik M, Ozuah PO, Franco K, Cohen R, Motlow F: Use of complementary therapy by adolescents with asthma. Arch Pediatr Adolesc Med 2002, 156:  1042-1044. H2.
Rhee DJ, Spaeth GL, Myers JS, Steinmann WC, Augsburger JJ, Shatz LJ et al.: Prevalence of the use of complementary and alternative medicine for glaucoma.  Ophthalmology 2002, 109: 438-443. H2, L3.
Rhi BY, Ha KS, Kim YS, Sasaki Y, Young D, Woon et al.: The health care seeking behavior of schizophrenic patients in 6 East Asian areas. Int J Soc Psychiatry 1995, 41: 190-209. H2, L3.
Riley-Doucet CK, Fouladbakhsh JM, Vallerand AH: Canadian and American self-treatment of pain: a comparison study. Rural Remote Health 2004, 4: 286. H2.
Ritchie CS, Gohmann SF, McKinney WP: Does use of CAM for specific health problems increase with reduced access to care? J Med Syst 2005, 29: 143-153. L2.
Ritchie MR: Use of herbal supplements and nutritional supplements in the UK: what do we know about their pattern of usage? Proc Nutr Soc 2007, 66: 479-482. L2, L3.
Rivera JO, Gonzalez-Stuart A, Ortiz M, Rodriguez JC, Anaya JP, Meza A: Guide for herbal product use by Mexican Americans in the largest Texas-Mexico border community. Tex Med 2006, 102: 46-56. L3.
Robinson N: Integrated traditional Chinese medicine. Complement Ther Clin Pract 2006, 12: 132-140. L2, N3.
Roehm CE, Tessema B, Brown SM: The role of alternative medicine in rhinology. Facial Plast Surg Clin North Am 2012, 20: 73-81. L3.
Rooney B, Fiocco G, Hughes P, Halter S: Provider attitudes and use of alternative medicine in a midwestern medical practice in 2001. WMJ 2001, 100: 27-31. L2.
Roy-Byrne PP, Bystritsky A, Russo J, Craske MG, Sherbourne CD, Stein MB: Use of herbal medicine in primary care patients with mood and anxiety disorders. Psychosomatics 2005, 46: 117-122. H2, L3.
Rutakumwa W, Krogman N: Women's health in rural Uganda: problems, coping strategies, and recommendations for change. Can J Nurs Res 2007, 39: 105-125. L3.
Ryder PT, Wolpert B, Orwig D, Carter-Pokras O, Black SA: Complementary and alternative medicine use among older urban African Americans: individual and neighborhood associations. J Natl Med Assoc 2008, 100: 1186-1192. H2, L3.
Saini NK, Gaur DR, Saini V, Lal S: Acute respiratory infections in children: a study of knowledge and practices of mothers in rural Haryana. J Commun Dis 1992, 24: 75-77. L3.
Samdup DZ, Smith RG, Il SS: The use of complementary and alternative medicine in children with chronic medical conditions. Am J Phys Med Rehabil 2006, 85: 842-846. L3.
Sanders H, Davis MF, Duncan B, Meaney FJ, Haynes J, Barton LL: Use of complementary and alternative medical therapies among children with special health care needs in southern Arizona. Pediatrics 2003, 111: 584-587. H2, L3.
Sandler AP, Chan LS: Mexican-American folk belief in a pediatric emergency room. Med Care 1978, 16: 778-784. L3.
Satow YE, Kumar PD, Burke A, Inciardi JF:  Exploring the prevalence of Ayurveda use among Asian Indians. J Altern Complement Med 2008, 14: 1249-1253. H2.
Satterthwaite D, Tacoli C: Seeking an understanding of poverty that recognizes rural-urban differences and rural-urban linkages. In Urban Livelihoods: A people-centred approach to reducing poverty. Edited by Radoki C, Lloyd-Jones T. London: Earthscan; 2002:52-70. H4.
Satyapan N, Patarakitvanit S, Temboonkiet S, Vudhironarit T, Tankanitlert J: Herbal medicine: affecting factors and prevalence of use among Thai population in Bangkok. J Med Assoc Thai 2010, 93 Suppl 6: S139-S144. H2.
Sauerborn R, Adams A, Hien M: Household strategies to cope with the economic cost of illness. Social Science and Medicine 1996, 43:291-301. L2, H2.
Sawalha AF: Complementary and alternative medicine (CAM) in Palestine: use and safety implications. J Altern Complement Med 2007, 13: 263-269. H2, L3.
Schaetti C, Khatib AM, Ali SM, Hutubessy R, Chaignat CL, Weiss MG: Social and cultural features of cholera and shigellosis in peri-urban and rural communities of Zanzibar. BMC Infect Dis 2010, 10: 339. H4, L3.
Scheinman D: Traditional medicine in Tanga today. IK Notes no. 51. Washington DC: World Bank; 2002. L3.
Schmidt K, Jacobs PA, Barton A: Cross-cultural differences in GPs' attitudes towards complementary and alternative medicine: a survey comparing regions of the UK and Germany. Complement Ther Med 2002, 10: 141-147. L2.
Sebit MB, Chandiwana SK, Latif AS, Gomo E, Acuda SW, Makoni F et al.: Quality of life evaluation in patients with HIV-I infection: the impact of traditional medicine in Zimbabwe. Cent Afr J Med 2000, 46: 208-213. H2, L3.
Sevilla-Dedieu C, Kovess-Masfety V, Haro JM, Fernandez A, Vilagut G, Alonso J: Seeking help for mental health problems outside the conventional health care system: results from the European Study of the Epidemiology of Mental Disorders (ESEMeD). Can J Psychiatry 2010, 55: 586-597. H2, L3.
Shalom T, Schiff E, Steiner M, Katz M, Ben-Arye E: [Integrating complementary medicine in oncology supportive care: assessment of patients' needs and expectations during chemotherapy]. Harefuah 2011, 150: 642-5, 689. L3.
Sharma N: Indigenous medication used by Himachali women to cure pregnancy discomforts. Indian Journal of Traditional Knowledge 2008, 7: 638-641. L3.
Sharma SC: Less - known medicinal uses of plants among the rural women of shahjahanpur district, u.p. Anc Sci Life 2000, 20: 29-32. L3.
Shen J, Oraka E: Complementary and alternative medicine (CAM) use among children with current asthma. Prev Med 2012, 54: 27-31. H2, L3.
Sheng-Ji P: Ethnobotanical approaches of traditional medicine studies: some experiences from Asia. Pharm Biol 2001, 39 Suppl 1: 74-79. L1.
Shih CC, Su YC, Liao CC, Lin JG: Patterns of medical pluralism among adults: results from the 2001 National Health Interview Survey in Taiwan. BMC Health Serv Res 2010, 10: 191. H2.
Shin DW: [Traditional medicine under Japanese rule after 1930s]. Uisahak 2003, 12: 110-128. N3.
Shinwari ZK, Qaiser M: Efforts on Conservation and Sustainable Use of Medicinal Plants of Pakistan. Pakistan Journal of Botany 2011, 43: 5-10. L1.
Shorofi SA, Arbon P: Complementary and alternative medicine (CAM) among hospitalised patients: an Australian study. Complement Ther Clin Pract 2010, 16: 86-91. H2.
Shorofi SA: Complementary and alternative medicine (CAM) among hospitalised patients: reported use of CAM and reasons for use, CAM preferred during hospitalisation, and the socio-demographic determinants of CAM users. Complement Ther Clin Pract 2011, 17: 199-205. H2.
Shriar AJ: Regional integration or disintegration? Recent road improvements in Petén, Guatemala: A review of preliminary economic, agricultural, and environmental impacts. Geoforum 2006, 37:104-112. N1.
Shumay DM, Maskarinec G, Kakai H, Gotay CC: Why some cancer patients choose complementary and alternative medicine instead of conventional treatment. J Fam Pract 2001, 50: 1067. L3.
Sibbritt DW, Adams J: Back pain amongst 8,910 young Australian women: a longitudinal analysis of the use of conventional providers, complementary and alternative medicine (CAM) practitioners and self-prescribed CAM. Clin Rheumatol 2010, 29: 25-32. L3.
Silano M, De VM, De VA, Silano V: The new European legislation on traditional herbal medicines: main features and perspectives. Fitoterapia 2004, 75: 107-116. N3.
Silva KT: Ayurveda, malaria and the indigenous herbal tradition in Sri Lanka. Soc Sci Med 1991, 33: 153-160. L3.
Singer J, Fisher K: The impact of co-option on herbalism: A bifurcation in epistemology and practice. Health Sociology Review 2007, 16: 18-26. L2.
Singh V, Raidoo DM, Harries CS: The prevalence, patterns of usage and people's attitude towards complementary and alternative medicine (CAM) among the Indian community in Chatsworth, South Africa. BMC Complement Altern Med 2004, 4: 3. H2, L3.
Singh YN, Ikahihifo T, Panuve M, Slatter C: Folk medicine in Tonga. A study of the use of herbal medicines for obstetric and gynaecological conditions and disorders. J Ethnopharmacol 1984, 12: 305-329. H3, H4, L2, L3.
Siti ZM, Tahir A, Farah AI, Fazlin SM, Sondi S, Azman AH et al.: Use of traditional and complementary medicine in Malaysia: a baseline study. Complement Ther Med 2009, 17: 292-299. H2.
Slader CA, Reddel HK, Jenkins CR, Armour CL, Bosnic-Anticevich SZ: Complementary and alternative medicine use in asthma: who is using what? Respirology 2006, 11: 373-387. L3.
Slater G, Tan B, Teh KC: Dietary supplementation practices of Singaporean athletes. Int J Sport Nutr Exerc Metab 2003, 13: 320-332. L3.
Smith TC, Ryan MA, Smith B, Reed RJ, Riddle JR, Gumbs GR et al.: Complementary and alternative medicine use among US Navy and Marine Corps personnel. BMC Complement Altern Med 2007, 7: 16. H2, L3.
Snow RW, Bronzan R, Roques T, Nyamawi C, Murphy S, Marsh K: The prevalence and morbidity of snake bite and treatment-seeking behaviour among a rural Kenyan population. Ann Trop Med Parasitol 1994, 88: 665-671. L3.
Sommers E, Porter K: Price elasticities for three types of CAM services: Experiences of a Boston Public Health Clinic. J Altern Complement Med 2006, 12: 85-90. H3.
Son AH: Modernisation of the system of traditional Korean medicine (1876-1990). Health Policy 1998, 44: 261-281. N3.
Son AH: Modernization of medical care in Korea (1876-1990). Soc Sci Med 1999, 49: 543-550. L2.
Soo I, Mah JK, Barlow K, Hamiwka L, Wirrell E: Use of complementary and alternative medical therapies in a pediatric neurology clinic. Can J Neurol Sci 2005, 32: 524-528. L3.
Souza CD de, Felfili JM: Uso de plantas medicinais na região de Alto Paraíso de Goiás, GO, Brasil. Acta Botanica Brasilica 2006, 20:135-142. N2.
Souza MH, Monteiro CA, Figueredo PM, Nascimento FR, Guerra RN: Ethnopharmacological use of babassu (Orbignya phalerata Mart) in communities of babassu nut breakers in Maranhao, Brazil. J Ethnopharmacol 2011, 133: 1-5. L3.
Srithi K, Balslev H, Wangpakapattanawong P, Srisanga P, Trisonthi C: Medicinal plant knowledge and its erosion among the Mien (Yao) in northern Thailand. J Ethnopharmacol 2009, 123: 335-342. H2.
Stangeland T, Dhillion SS, Reksten H: Recognition and development of traditional medicine in Tanzania. Journal of Ethnopharmacology 2008, 117: 290-299. N1, N3.
Steinhoff B: Laws and regulation on medicinal and aromatic plants in Europe. Acta Horticulturae 2005, 678:13-22. N3.
Steel A, Adams J: The role of naturopathy in pregnancy, labour and post-natal care: broadening the evidence-base. Complement Ther Clin Pract 2011, 17: 189-192. H2, L3.
Stewart KM: The African cherry (Prunus africana): can lessons be learned from an over-exploited medicinal tree?  J Ethnopharmacol 2003, 89: 3-13. L1.
Sullivan LV, Hicks P, Salazar G, Robinson CK: Patient beliefs and sense of control among Spanish-speaking patients with diabetes in northeast Colorado. J Immigr Minor Health 2010, 12: 384-389. H2, L3.
Sumngern C, Azeredo Z, Subgranon R, Matos E, Kijjoa A: The perception of the benefits of herbal medicine consumption among the Thai elderly. J Nutr Health Aging 2011, 15: 59-63. H2.
Sunderlin WD, Hatcher J, Liddle M: From exclusion to ownership? Washington DC: Rights and Resources Initiative; 2008. L1.
Super EA, Kemper KJ, Woods C, Nagaraj S: Cranberry use among pediatric nephrology patients. Ambul Pediatr 2005, 5: 249-252. H2, L3.
Tabuti JR: Herbal medicines used in the treatment of malaria in Budiope county, Uganda. J Ethnopharmacol 2008, 116: 33-42. L3.
Tahzib F, Daniel SO: Traditional medicine and the modern medical curriculum. Lancet 1986, 2: 203-204. N3.
Tait EM, Laditka SB, Laditka JN, Nies MA, Racine EF: Use of Complementary and Alternative Medicine For Physical Performance, Energy, Immune Function, and General Health Among Older Women and Men in the United States. Journal of Women & Aging 2012, 24: 23-43. H2, L3.
Tapsoba H, Deschamps J-P: Use of medicinal plants for the treatment of oral diseases in Burkina Faso. Journal of Ethnopharmacology 2006, 104:68-78. H2, L3.
Tarhan O, Alacacioglu A, Somali I, Sipahi H, Zencir M, Oztop I et al.: Complementary-alternative medicine among cancer patients in the western region of Turkey. J BUON 2009, 14: 265-269. H2, L3.
Tas F, Ustuner Z, Can G, Eralp Y, Camlica H, Basaran M et al.: The prevalence and determinants of the use of complementary and alternative medicine in adult Turkish cancer patients. Acta Oncol 2005, 44: 161-167. H2.
Teixeira ER, de Nogueira JF: [The popular use of therapeutic herbs in body care]. Rev Gaucha Enferm 2005, 26: 231-241. H2.
Teklehaymanot T, Giday M, Medhin G, Mekonnen Y: Knowledge and use of medicinal plants by people around Debre Libanos monastery in Ethiopia. J Ethnopharmacol 2007, 111: 271-283. H2.
Thomas K, Coleman P: Use of complementary or alternative medicine in a general population in Great Britain. Results from the National Omnibus survey. J Public Health (Oxf) 2004, 26: 152-157. H2.
Thomas KJ, Nicholl JP, Coleman P: Use and expenditure on complementary medicine in England: a population based survey. Complement Ther Med 2001, 9: 2-11. H2.
Tian HH, Ong WS, Tan CL: Nutritional supplement use among university athletes in Singapore. Singapore Med J 2009, 50: 165-172. L3.
Tillisch K: Complementary and alternative medicine for gastrointestinal disorders. Clin Med 2007, 7: 224-227. L3.
Tindle HA, Davis RB, Phillips RS, Eisenberg DM: Trends in use of complementary and alternative medicine by US adults: 1997-2002. Altern Ther Health Med 2005, 11: 42-49. H2. 
Tobgay T, Dorji T, Pelzom D, Gibbons RV: Progress and delivery of health care in Bhutan, the Land of the Thunder Dragon and Gross National Happiness. Trop Med Int Health 2011, 16: 731-736. N3.
Tokem Y: [The use of complementary and alternative treatment in patients with asthma]. Tuberk Toraks 2006, 54: 189-196. L3.
Toprak D, Demir S: Treatment choices of hypertensive patients in Turkey. Behav Med 2007, 33: 5-10. H2, L3.
Torri MC: Fostering traditional health systems and ethnomedicine practices through a holistic approach: a pioneering community strategy from Southern India. Int Q Community Health Educ 2009, 30: 3-20. L1.
Trigazis L, Tennankore D, Vohra S, Katzman DK: The use of herbal remedies by adolescents with eating disorders. Int J Eat Disord 2004, 35:  223-228. H2.
Trotter RT: Folk remedies as indicators of common illnesses: examples from the United States-Mexico border. J Ethnopharmacol 1981, 4: 207-221. L3.
Tsabang N, Fokou PVT, Tchokouaha LRY, Noguem B, Bakarnga-Via I, Nguepi MSD et al.: Ethnopharmacological survey of Annonaceae medicinal plants used to treat malaria in four areas of Cameroon. Journal of Ethnopharmacology 2012, 139: 171-180. L3.
Tsey K: Traditional medicine in contemporary Ghana: a public policy analysis. Soc Sci Med 1997, 45: 1065-1074. L2, N3.
Tsutani K, Takuma H: [Regulatory sciences in herbal medicines and dietary supplements]. Yakugaku Zasshi 2008, 128: 867-880. N3.
Tumwesigye O: Bumetha Rukararwe: integrating modern and traditional health care in southwest Uganda. J Altern Complement Med 1996, 2: 373-376. L2.
Ulmer EM, Hohmann U, Linhart M, Kohan D, Saller R: [Using interaction-intensive therapeutic nursing measures and "home remedies" in nursing care. An explorative study as a plea for integrated nursing practice]. Pflege 2001, 14: 191-205. H2.
UNAIDS: Collaboration with traditional healers in HIV/AIDS prevention and care in sub-Saharan Africa – a literature review. Geneva: UNAIDS; 2000. L2.
Vaiva G, Jehel L, Cottencin O, Ducrocq F, Duchet C, Omnes C et al.: [Prevalence of trauma-related disorders in the French WHO study: Sante mentale en population generale (SMPG)]. Encephale 2008, 34: 577-583. L3.
Vallerand AH, Fouladbakhsh JM, Templin T: Self-treatment of pain in a rural area. J. Rural Health 2004, 20: 166-172. L2.
van Andel T, Havinga R: Sustainability aspects of commercial medicinal plant harvesting in Suriname. Forest Ecology and Management 2008, 256: 1540-1545. L1.
van Andel T, Westers P: Why Surinamese migrants in the Netherlands continue to use medicinal herbs from their home country. Journal of Ethnopharmacology 2010, 127: 694-701. H2.
van Andel T, Myren B, van Onselen S: Ghana's herbal market. Journal of Ethnopharmacology 2012, 140: 368-378. L3.
van der Sluijs CP, Bensoussan A, Liyanage L, Shah S: Women's health during mid-life survey: the use of complementary and alternative medicine by symptomatic women transitioning through menopause in Sydney. Menopause 2007, 14: 397-403. L3.
van Tilburg MA, Palsson OS, Levy RL, Feld AD, Turner MJ, Drossman DA et al.: Complementary and alternative medicine use and cost in functional bowel disorders: a six month prospective study in a large HMO. BMC Complement Altern Med 2008, 8: 46. H2.
van AT, Westers P: Why Surinamese migrants in the Netherlands continue to use medicinal herbs from their home country. J Ethnopharmacol 2010, 127: 694-701. H2, L3.
van EP: To strengthen and refresh: herbal therapy in Southeast Asia. Soc Sci Med 1988, 27: 751-759. L2.
	Vandebroek I, Calewaert JB, De jS, Sanca S, Semo L, Van DP et al.: Use of medicinal plants and pharmaceuticals by indigenous communities in the Bolivian Andes and Amazon. Bull World Health Organ 2004, 82: 243-250. H2, H3.
Vandebroek I, Damme PV, Puyvelde LV, Arrazola S, Kimpe ND: A comparison of traditional healers' medicinal plant knowledge in the Bolivian Andes and Amazon. Social Science and Medicine 2004, 59:837-849. L2.
Vandebroek I, Thomas E, Sanca S, Van DP, Puyvelde LV, De KN: Comparison of health conditions treated with traditional and biomedical health care in a Quechua community in rural Bolivia. J Ethnobiol Ethnomed 2008, 4: 1. L3.
Vandebroek I, Balick MJ, Ososki A, Kronenberg F, Yukes J, Wade C et al.: The importance of botellas and other plant mixtures in Dominican traditional medicine. J Ethnopharmacol 2010, 128: 20-41. H2, L3.
Vandebroek I, Balick MJ: Globalization and loss of plant knowledge: challenging the paradigm. PLoS One 2012, 7: e37643. H4.
Vanwambeke SO, Lambin EF, Eichhorn MP, Flasse SP, Harbach RE, Oskam L, Somboon P, Beers SV, Benthem BHBV, Walton C, Butlin RK: Impact of land-use change on dengue and malaria in Northern Thailand. EcoHealth 2007, 4:37-51. N5.
Varga CA, Veale DJ: Isihlambezo: utilization patterns and potential health effects of pregnancy-related traditional herbal medicine. Soc Sci Med 1997, 44: 911-924. H4.
Vlieger AM, van de Putte EM, Hoeksma H: [The use of complementary and alternative medicine in children at a general paediatric clinic and parental reasons for use]. Ned Tijdschr Geneeskd 2006, 150: 625-630. H2, L3.
Vlieger AM, van VM, Jong MC: Attitudes toward complementary and alternative medicine: a national survey among paediatricians in the Netherlands. Eur J Pediatr 2011, 170: 619-624. L2.
Voeks RA, Sercombe P: The scope of hunter-gatherer ethnomedicine. Social Science and Medicine 2000, 51:679-690. L2,
Vollan B, Ostrom E: Cooperation and the Commons. Science 2010, 330:923-924. L1.
Volpato G, Godinez D: Ethnobotany of Pru, a traditional Cuban refreshment. Economic Botany 2004, 58: 381-395. L1.
Wagner PJ, Jester D, LeClair B, Taylor AT, Woodward L, Lambert J: Taking the edge off: why patients choose St. John's Wort. J Fam Pract 1999, 48: 615-619. L3.
Wahlberg A: Bio-politics and the promotion of traditional herbal medicine in Vietnam. Health (London) 2006, 10: 123-147. N3.
Walker AR, Walker BF, Manetsi B, Tsotetsi NG, Segal I: Appendicitis in Soweto, South Africa: traditional healers and hospitalization. Journal of the Royal Society for the Promotion of Health 1989, 109:190-192. N1.
Wambui T, Ek AC, Alehagen S: Cognizance of sexually transmitted infections among low-income men in western Kenya. Sex Reprod Healthc 2011, 2: 169-173. H2, L3.
Wang D, Liu S, Liang Y: [A study on producing areas of Chinese flos Dendranthematis]. Zhongguo Zhong Yao Za Zhi 1999, 24: 522-5, 573. L1.
Wang SM, Peloquin C, Kain ZN: Attitudes of patients undergoing surgery toward alternative medical treatment. Journal of Alternative and Complementary Medicine 2002, 8: 351-356. H2, L3.
Wangchuk P, Wangchuk D, Aagaard-Hansen J: Traditional Bhutanese medicine (gSo-BA Rig-PA): an integrated part of the formal health care services. Southeast Asian J Trop Med Public Health 2007, 38: 161-167. L2, N3.
Wayland C: The failure of pharmaceuticals and the power of plants: medicinal discourse as a critique of modernity in the Amazon. Social Science & Medicine 2004, 58: 2409-2419. H4.
Wehi PM, Wehi WL: Traditional plant harvesting in contemporary fragmented and urban landscapes. Conservation Biology 2010, 24:594-604. H2, H4.
White AR, Ernst E: Economic analysis of complementary medicine: a systematic review. Complementary Therapies in Medicine 2000, 8:111-118. H2.
White B, Knox L, Zepeda M, Mull D, Nunez F: Impact of immigration on complementary and alternative medicine use in Hispanic patients. J Am Board Fam Med 2009, 22: 337-338. H2, L3, L4.
WHO: Legal status of traditional medicine and complementary/alternative medicine: a worldwide review. Geneva: World Health Organisation; 2001. N3.
WHO: WHO Strategy for Traditional Medicine 2002-2005, Regional Committee, 55th Session, SEA/RC55/13, 2002. I4.
Widmer M, Herren S, Donges A, Marian F, Busato A: Complementary and conventional medicine in Switzerland: comparing characteristics of general practitioners. Forsch Komplementmed 2006, 13: 234-240. L2.
Wijesekera RO: Is there an industrial future for phytopharmaceutical drugs? An outline of UNIDO programmes in the sector. J Ethnopharmacol 1991, 32: 217-224. I4.
Wilcox ML, Bodeker G: Traditional herbal medicines for malaria. British Medical Journal 2004, 329:1156-1159. L3.
Williams VL, Balkwill K, Witkowski ETF: Muthi traders on the Witwatersrand, South Africa - an urban mosaic. South African Journal of Botany 1997, 63: 378-381. H3.
Williamson AT, Fletcher PC, Dawson KA: Complementary and alternative medicine. Use in an older population. J Gerontol Nurs 2003, 29: 20-28. H2.
Wilson KM, Klein JD, Sesselberg TS, Yussman SM, Markow DB, Green AE et al.: Use of complementary medicine and dietary supplements among U.S. adolescents.  J Adolesc Health 2006, 38: 385-394. H2.
Winkler AS, Mayer M, Ombay M, Mathias B, Schmutzhard E, Jilek-Aall L: Attitudes towards African traditional medicine and Christian spiritual healing regarding treatment of epilepsy in a rural community of northern Tanzania. Afr J Tradit Complement Altern Med 2010, 7: 162-170. H2, L2.
Winnick TA: Medical doctors and complementary and alternative medicine: the context of holistic practice. Health 2006, 10:149-173. L2.
Wong DFK, He XS: Schizophrenia literacy among Chinese in Shanghai, China: a comparison with Chinese-speaking Australians in Melbourne and Chinese in Hong Kong. Australian and New Zealand Journal of Psychiatry 2011, 45: 524-531. H2.
Wong DFK, Lam AYK, Poon A, Chow AYM: Gender differences in mental health literacy among Chinese-speaking Australians in Melbourne, Australia. International Journal of Social Psychiatry 2012, 58: 178-185. H2, H4, L3.
Wong FK, Lam YK, Poon A: Depression literacy among Australians of Chinese-speaking background in Melbourne, Australia. BMC Psychiatry 2010, 10: 7. H2, L3.
Wong FK, Lam YK, Poon A: Knowledge and preferences regarding schizophrenia among Chinese-speaking Australians in Melbourne, Australia. Soc Psychiatry Psychiatr Epidemiol 2010, 45: 865-873. H2, L3.
Wong LC, Chan E, Tay S, Lee KM, Back M: Complementary and alternative medicine practices among Asian radiotherapy patients. Asia Pac J Clin Oncol 2010, 6: 357-363. H2, L3.
Wong LKH, Jue P, Lam A, Yeung W, Cham-Wah Y, Birtwhistle R: Chinese herbal medicine and acupuncture - How do patients who consult family physicians use these therapies? Canadian Family Physician 1998, 44: 1009-1015. H2, L3, L4.
Wood MJ, Stewart RL, Merry H, Johnstone DE, Cox JL: Use of complementary and alternative medical therapies in patients with cardiovascular disease. American Heart Journal 2003, 145: 806-812. L3.
Woodward A, Hales S, Litidamu N: Protecting human health in a changing world: the role of social and economic development. Bulletin of the World Health Organization 2000, 78:1148-1155. N3.
Wulandari LP, Klinken WA: Beliefs, attitudes and behaviours of pregnant women in Bali. Midwifery 2011, 27: 867-871. L3.
Xiao PG, Chen SL: [Establishment of the national macro-management system on Chinese medicinal resource--base of the modernization for Chinese medicine]. Zhongguo Zhong Yao Za Zhi 2003, 28: 4-6. N1.
Xiong F, Ma Y, Yan Z, Chen X, Zhu M, Chen R: [Investigation and protection for endangered Coptis deltoidea]. Zhongguo Zhong Yao Za Zhi 2011, 36: 968-972. L1.
Xu J, Yang Y, Li Z, Tashi N, Sharma R, Fang J: Understanding land use, livelihoods, and health transitions among Tibetan nomads: A case from Gangga Township, Dingri County, Tibetan Autonomous Region of China. EcoHealth 2008, 5:104-114. N7.
Yadav SP, Sharma RC, Joshi V: Treatment seeking behaviour of malaria patients in desert part of Rajasthan, India. J Commun Dis 2007, 39: 57-64. L3.
Yates JS, Mustian KM, Morrow GR, Gillies LJ, Padmanaban D, Atkins JN et al.: Prevalence of complementary and alternative medicine use in cancer patients during treatment. Support Care Cancer 2005, 13: 806-811. H2, L3.
Yeh GY, Eisenberg DM, Davis RB, Phillips RS: Use of complementary and alternative medicine among persons with diabetes mellitus: results of a national survey. Am J Public Health 2002, 92: 1648-1652. H2, L3.
Yeh GY, Davis RB, Phillips RS: Use of complementary therapies in patients with cardiovascular disease. Am J Cardiol 2006, 98: 673-680. H2, L3.
Yildirim Y, Tinar S, Yorgun S, Toz E, Kaya B, Sonmez S et al.: The use of complementary and alternative medicine (CAM) therapies by Turkish women with gynecological cancer. Eur J Gynaecol Oncol 2006, 27: 81-85. H2.
Yoo TW, Kim BI, Kim JB, Kim DJ, Kim JW, Baik SK et al.: [The survey for the actual condition of drug medication and development of health care cost associated with toxic liver injury in Korean: a multicenter study for the detection and the development of nationwide reporting system of toxic liver injury]. Korean J Hepatol 2007, 13: 34-43. H2.
Yussman SM, Ryan SA, Auinger P, Weitzman M: Visits to complementary and alternative medicine providers by children and adolescents in the United States. Ambul Pediatr 2004, 4: 429-435. H2, L2.
Zaffani S, Cuzzolin L, Benoni G: Herbal products: behaviors and beliefs among Italian women. Pharmacoepidemiol Drug Saf 2006, 15: 354-359. H2, L3.
Zank S, Hanazaki N: Exploring the Links between Ethnobotany, Local Therapeutic Practices, and Protected Areas in Santa Catarina Coastline, Brazil. Evid Based Complement Alternat Med 2012, 2012: 563570. H2.
Zhang EY: Switching between traditional chinese medicine and viagra: cosmopolitanism and medical pluralism today. Med Anthropol 2007, 26: 53-96. L2.
Zhang W, Zhang RX: [The motive characteristics and pattern of manifestation of Chinese medicinal herb during the period of "Cultural Revolution"]. Zhonghua Yi Shi Za Zhi 2009, 39: 25-29. N3.
Zhang Y, Peck K, Spalding M, Xu T, Ragain M: A study to examine the attitudes, knowledge, and utilization of CAM by primary care professional in West Texas. Complement Ther Med 2010, 18: 227-232. H2.
Zhang YT, Fein EB, Fein SB: Feeding of Dietary Botanical Supplements and Teas to Infants in the United States. Pediatrics 2011, 127: 1060-1066. H2.
Zhong GY, Qin SY, Wang CH, Liu X, Gu R: [Study on methods of dynamic monitoring of traditional Chinese medicine resources]. Zhongguo Zhong Yao Za Zhi 2008, 33: 2570-2574. L1.
Zhou YQ, Chen SL, Zhao RH, Xie CX, Li Y: [Study on application of low altitude remote sensing to Chinese herb medicinal sustainable utilization]. Zhongguo Zhong Yao Za Zhi 2008, 33: 977-979. L1.
Zhu X, Qi X, Hao J, Huang Z, Zhang Z, Xing X et al.: Pattern of drug use during the first trimester among Chinese women: data from a population-based cohort study.  Eur J Clin Pharmacol 2010, 66: 511-518. H2, L3.
Zuckerman MJ, Guerra LG, Drossman DA, Foland JA, Gregory GG: Health-care-seeking behaviors related to bowel complaints. Hispanics versus non-Hispanic whites. Dig Dis Sci 1996, 41: 77-82. H2.
Zuzak TJ, Zuzak-Siegrist I, Rist L, Staubli G, Simoes-Wust AP: Medicinal systems of complementary and alternative medicine: a cross-sectional survey at a pediatric emergency department. J Altern Complement Med 2010, 16: 473-479. L3.
